# Phylogenetic Inference and Ancestral Character Reconstruction of Diphyllobothriid Tapeworms (Cestoda: Diphyllobothriidae)

**DOI:** 10.3390/ani16132084

**Published:** 2026-07-06

**Authors:** Sisi Ru, Yanyan Zhou, Haijun Jiang, Huiran Zhang, Hongying Zhang, Xi Zhang

**Affiliations:** Department of Parasitology, School of Basic Medical Sciences, Zhengzhou University, Zhengzhou 450001, China; russ248@163.com (S.R.); zhouyy2025@zzu.edu.cn (Y.Z.); jhj2021@163.com (H.J.); zhanghuiran2022@163.com (H.Z.); m17658156816@163.com (H.Z.)

**Keywords:** ancestral traits, divergence time, Diphyllobothriidae, mitogenome, phylogeny

## Abstract

Some groups of Diphyllobothriid tapeworms can cause foodborne or waterborne taeniasis, yet their systematics and evolution remain poorly understood. Based on mitochondrial genome data, this study reconstructed the phylogenetic trees of 11 cestode orders using maximum likelihood and Bayesian methods, clarified the valid species of *Spirometra*, traced the evolutionary history of four key ecological traits, and estimated the divergence times of major groups. Phylogenetic analyses supported Diphyllobothriidea and Bothriocephalidea as distinct clades, with Diphyllobothriidea forming a sister group to Haplobothriidea. In addition, three novel types of mitochondrial gene arrangements were identified in tapeworms, and variations in these arrangement types appear to correspond to changes in proglottid morphology. Species validation has shown that the available *Spirometra* mitogenomes represent only four valid species. Phylogenetic analysis identified two genetic lineages within *S. mansoni*, and a comparison of their representative sequences showed that the differences mainly lie in 12 PCGs, 7 tRNAs, 2 rRNAs, and 2 mNCRs. Ancestral character reconstruction suggested that tapeworms likely originated from freshwater fish hosts, with gradual adaptation to terrestrial hosts representing the main pathway of diversification, whereas transitions to marine environments were independent evolutionary events occurring in only a few groups. Molecular clock analyses indicated that tapeworm diversification began in the mid-to-late Oligocene, with episodic species radiations in different groups closely linked to host diversification and climatic changes. Notably, most groups within the family Diphyllobothriidae radiated during the Pleistocene, whereas *Spirometra* radiated during the Pliocene, coinciding with the origin of human-specialized cestodes. This study clarifies the phylogenetic position of diphyllobothriid tapeworms, infers the evolutionary patterns of ancestral traits across different tapeworm groups, and provides a theoretical basis for elucidating the origin and species diversity of tapeworms, as well as for assessing the transmission risk of pathogenic species.

## Simple Summary

Some diphyllobothriid tapeworms can cause foodborne or waterborne infections in humans and animals, but their evolutionary relationships are still unclear. In this study, we used mitochondrial genomes to build evolutionary trees for 11 tapeworm groups and perform ancestral character reconstruction. We found that the order Diphyllobothriidea is a distinct lineage and identified new patterns in the organization of mitochondrial genes. By validating species names, we showed that only four valid species of the genus *Spirometra* exist, and *S. mansoni* contains two hidden genetic types. Our results suggest that tapeworms originally evolved in freshwater fish and then adapted to land animals multiple times. Returns to the sea happened rarely and independently in a few groups. Tapeworm diversification began in the mid-to-late Oligocene, with most diphyllobothriid tapeworms radiating during the Pleistocene, but *Spirometra* diversified earlier (Pliocene), around the time when human-specialized tapeworms appeared. This work helps us understand how tapeworms evolved, where they came from, and how to assess the risk of infections caused by these parasites.

## 1. Introduction

Diphyllobothriid tapeworms (Diphyllobothriidea: Diphyllobothriidae) are globally distributed cestodes [1]. They range in length from a few centimetres to tens of metres and possess a scolex with bothria. Their life cycle typically involves three hosts: copepods as first intermediate hosts, fish as second intermediate hosts (*Spirometra* species mainly use amphibians and reptiles), and marine or terrestrial mammals and fish-eating birds as definitive hosts [2]. Some groups are important foodborne and waterborne zoonotic pathogens. Adults of *Diphyllobothrium* parasitize the human intestine, causing diphyllobothriasis, which may lead to gastrointestinal discomfort, vitamin B12 deficiency, and megaloblastic anaemia [3]. The larvae (spargana) of *Spirometra* can invade multiple human organs and tissues, causing sparganosis, primarily manifesting as larva migrans, which can result in local tissue damage, blindness, limb paralysis, and even death [4]. Furthermore, *Sparganum proliferum* can continuously proliferate within the human body, leading to nodular abscesses in multiple organs and causing more severe harm [5]. In recent years, factors such as changes in dietary habits, insufficient quarantine measures, and frequent international travel and trade have increased the risk of infection with these parasites, with related diseases showing an accelerated global spread [6]. However, the taxonomic status of the diphyllobothriid tapeworms remains unclear, as research on their patterns of genetic evolution is still very limited [1,2,7].

The order Diphyllobothriidea was formerly classified under the order Pseudophyllidea but was later established as an independent order based on ribosomal RNA data [8,9,10]. However, since this taxonomic revision, phylogenetic studies specifically targeting this group have been relatively scarce, and most studies have focused on Cyclophyllidea, with insufficient coverage of rare groups such as Tetraphyllidea, Proteocephalidea, and Rhinebothriidea [11,12,13]. Although recent studies have attempted to include multiple orders, the number of representative species for some groups is limited, making it difficult to fully reflect true phylogenetic relationships [14]. Regarding the family Diphyllobothriidae, although studies have used markers such as *cox*1, *nad*1, *cytb*, and ribosomal RNA, the effective informative sites provided by single genes or a few concatenated genes are limited [7,15,16,17]. In contrast, mitochondrial genomes (mitogenomes) contain a richer set of evolutionarily informative sites, providing a more reliable basis for phylogenetic inference. Based on this, various researchers have used mitogenomic data to resolve phylogenetic relationships among some groups. For example, within the Diphyllobothriidea, the family Cephalochlamydidae represented the earliest diverging lineage, while Solenophoridae and Diphyllobothriidae formed a sister group relationship [2]. Within the Diphyllobothriidae, *Diplogonoporus* is regarded as a synonym of *Diphyllobothrium*, and the two form a monophyletic group that is sister to *Spirometra* [18]; *Digramma* is regarded as a synonym of *Ligula*, and together, they form the sister group to *Dibothriocephalus* [12]. The genus *Spirometra* comprises 46 nominal species, yet only seven are currently recognized [15,19]. Among these, *S. erinaceieuropaei* was restricted to Europe, whereas only *S. mansoni* and *S. asiana* (the latter known only from Japan and Korea) were confirmed in Asia [20,21]. Although *cox*1, *cytb*, and SSR markers have suggested the presence of two genetic clades of *S. mansoni* in China, the differences between these two clades at the mitogenomic level remain unclear [22,23]. To date, 22 *Spirometra* mitogenomes have been reported, but most are from early studies with isolated single-genome reports biased in geographic origin and host taxa, leaving interspecific relationships and valid names unclear [18,24,25]. Therefore, we sequenced two strains of Chinese *S. mansoni* representing different genetic lineages and integrated them with relevant published sequences to clarify their divergence patterns and taxonomic status. Furthermore, Fraija-Fernández et al. conducted the first ancestral character reconstruction for the Diphyllobothriidea, inferring that it likely originated in freshwater environments and more likely used reptiles as definitive hosts, laying a foundation for further understanding the evolutionary history of diphyllobothriid tapeworms [2]. However, with the increasing number of mitogenomic sequences in public databases, their study can be built upon, such as by incorporating more representative species within a larger phylogenetic framework to test the systematic stability of diphyllobothriid tapeworms and performing more extensive ancestral character reconstructions to gain a more comprehensive understanding of their evolutionary history.

Therefore, we aimed to utilize all publicly available mitogenomic data of diphyllobothriid tapeworms and related groups to conduct more comprehensive phylogenetic reconstruction, ancestral state inference, and divergence time estimation. Specifically, the main objectives of this study include the following: (1) reconstructing the phylogenetic relationships of diphyllobothriid tapeworms and related groups, exploring the interrelationships among different lineages, and focusing on the *Spirometra* to revise its valid species; and (2) revealing the ancestral states and evolutionary trajectories of different ecological traits of cestodes, including host habitat, number of hosts, second intermediate host type, and definitive host type, and estimating the divergence times of various groups using molecular clock methods. These analyses aim to further elucidate the drivers of cestode species diversity and explore the patterns of their origin and evolution.

## 2. Materials and Methods

### 2.1. Data Collection

In this study, 57 diphyllobothriid mitogenomic sequences were obtained from GenBank (as of 30 June 2025). After evaluation and verification, 44 sequences were retained: 22 from *Spirometra*, 6 from *Ligula*, 6 from *Dibothriocephalus*, 5 from *Diphyllobothrium*, 4 from *Schistocephalus*, and 1 from *Adenocephalus*. Additionally, two Chinese geographical strains of *S. mansoni* representing distinct genetic lineages were selected for mitogenome sequencing: AH_LA6 (from *Pelophylax nigromaculatus*, Anhui) and GX_WZ2 (from *Fejervarya limnocharis*, Guangxi) [22,23,26]. To examine broader phylogenetic relationships, mitogenomes were also collected from other cestode groups: Diphyllobothriidea (2 isolates each from Cephalochlamydidae and Scyphocephalidae), as well as from Bothriocephalidea (11), Caryophyllidea (5), Cyclophyllidea (50), Haplobothriidea (1), Nippotaeniidea (1), Proteocephalidea (2), Rhinebothriidea (16), Spathebothriidea (1), Tetraphyllidea (4), and Trypanorhyncha (1). Four trematode species (*Schistosoma japonicum*, *S. mekongi*, *S. mansoni*, and *S. haematobium*) were used as outgroups. Detailed sequence information is provided in Supplementary Table S1.

### 2.2. Mitogenome Sequencing and Annotation

Genomic DNA was extracted using the EasyPure Genomic DNA Kit (TransGen, Beijing, China). The sequencing libraries were prepared with the Nextera XT DNA Library Prep Kit (Illumina, San Diego, CA, USA) and sequenced on an Illumina NovaSeq 6000 platform. The raw reads were quality-filtered with fastp v0.19.7 [27]. Mitogenome assembly was performed using SPAdes v3.14.1 [28]. Annotation was conducted using the MITOS web server (http://mitos.bioinf.uni-leipzig.de/index.py, accessed on 8 June 2025) to identify 12 protein-coding genes (PCGs), 2 rRNA genes (*rrnL* and *rrnS*), and 22 tRNA genes. PCGs were verified using the ORF Finder tool (https://www.ncbi.nlm.nih.gov/orffinder/, accessed on 8 June 2025). The two major noncoding regions (mNCRs) were identified via BLAST v2.16.0+ (https://blast.ncbi.nlm.nih.gov/, accessed on 8 June 2025) alignment against the *S. mansoni* sequence (AB374543). Circular mitogenome maps were generated with Organellar Genome DRAW v1.2 (https://chlorobox.mpimp-golm.mpg.de/OGDraw.html, accessed on 12 June 2025). The secondary structures of the tRNAs were predicted with ViennaRNA v2.6.4 [29]. Tandem repeat sequences were detected with the Tandem Repeats Finder v4.09 (https://tandem.bu.edu/trf/trf.html, accessed on 18 June 2025). The secondary structures of the mNCR sequences were predicted with RNAstructure v6.3 [30]. Nucleotide composition, amino acid usage, and relative synonymous codon usage (RSCU) were calculated using PhyloSuite v1.2.3 [31]. Mitochondrial gene rearrangement events within Cestoda were inferred using the CREx program in Galaxy v0.1.8 [32]. The newly sequenced mitogenomes were deposited in the NCBI under accession numbers PX897701 and PX897702.

### 2.3. Phylogenetic Analysis

The 12 PCGs and 2 rRNA genes were aligned separately using MAFFT v7.313 [33]. Ambiguously aligned sites in PCGs and rRNAs were trimmed with Gblocks v0.91 and trimAl v1.2, respectively [34,35]. Nucleotide substitution saturation was assessed by plotting the number of transitions (Ti) against transversions (Tv) relative to corrected genetic distances in DAMBE v7.3.32 [36]. The trimmed alignments were concatenated into a single matrix using PhyloSuite v1.2.3 [31]. Phylogenetic trees were reconstructed from the concatenated nucleotide sequences of the 12 PCGs using both maximum likelihood (ML) and Bayesian inference (BI) methods. The optimal partition scheme and substitution models were determined with ModelFinder v2.2.0 under the Bayesian information criterion (BIC) [37]. The selected models and partitioning schemes for each analysis are detailed in Supplementary Table S2. The ML tree was constructed with IQ-TREE v2.2.0, and branch support was assessed using 1000 ultrafast bootstrap replicates [38]. The BI analysis was performed using MrBayes v3.2.7, with two independent Markov chain Monte Carlo (MCMC) runs of four chains for 10 million generations, with sampling every 500 generations [39]. The first 25% of the samples were discarded as burn-in, and convergence was assumed when the average standard deviation of split frequencies (ASDSF) fell below 0.01. The final trees were visualized and annotated using iTOL v6.1.1 [40].

### 2.4. Species Delimitation Within Spirometra

Based on the trimmed nucleotide sequences of the 12 PCGs and 2 rRNAs, the overall nucleotide diversity (*π*) of *Spirometra* was calculated using DnaSP v5 [41]. The diversity of individual genes was compared through sliding window analysis (window size: 100 bp; step size: 25 bp). The four genes with the highest *π* values were selected for species delimitation analysis using Assemble Species by Automatic Partitioning (ASAP) on 24 *Spirometra* sequences [42]. ASAP is an automated method for species delimitation that uses a scoring system to identify potential species boundaries directly from pairwise genetic distances of single-locus data, with lower scores indicating better-supported partitions. Analyses were run on the ASAPy v0.1.2 under default settings. This method has been widely used to delimit similar species in other taxa, such as insects, nematodes, and acanthocephalans [43,44,45]. Additionally, Bayesian phylogenetic trees were reconstructed separately from each of the four selected genes, following the methodology described above.

### 2.5. Ancestral Character Estimation

Ancestral state reconstruction for four traits (host habitat, number of hosts in the life cycle, type of second intermediate host, and type of definitive host) was performed in PastView v46 under the F81 model [46]. Ambiguous ancestral node states are visualized as pie charts showing their proportional likelihoods. Host habitat traits were divided into three states (“terrestrial”, “freshwater”, and “marine”) based on the habitats of the second intermediate and definitive hosts. The number of host traits was classified into three states (“one-host”, “two-host”, and “three-host”). *Rodentolepis nana*, which can complete its entire life cycle within the intestinal tract of a host, was designated “one host”. For species in Solenophoridae (*Bothridium pithonis* and *Duthiersia expansa*) and Proteocephalidae (*Testudotaenia* sp.), whose life cycles are unclear, the “three-host” state was assigned because of the feeding habits of their definitive hosts (reptiles) [2,11]. The second intermediate host trait was divided into four states (“absent”, “unknown”, “tetrapods”, and “fish”). Definitive host traits were classified into seven states (“fish”, “amphibians”, “reptiles”, “birds”, “terrestrial mammals”, “cetaceans”, and “pinnipeds”). The definitive host types for Diphyllobothriidea are the most complex, involving all groups except fish. The definitive host types for Cyclophyllidea are also relatively diverse and include amphibians (*Cylindrotaenia japonica* in Nematotaeniidae), birds (*Cloacotaenia megalops* and *Drepanidotaenia lanceolata* in Hymenolepididae; *Paruterina candelabraria* and *Cladotaenia vulturi* in Paruterinidae), and terrestrial mammals (the remaining species in Cyclophyllidea). The states of *S. proliferum* are defined in reference to species in *Spirometra* [15]. The full trait classifications for each species are provided in Supplementary Table S3.

### 2.6. Divergence Time Estimation

Divergence times among major cestode lineages were estimated using BEAST v2.7.7 based on the concatenated sequences of 12 PCGs and 2 rRNAs under an optimized relaxed molecular clock model [47]. The best partition scheme and substitution models were selected with ModelFinder v2.2.0, and a Yule model was used as the tree prior. MCMC analysis was run for 50,000,000 generations, with sampling every 5000 generations. Four calibration points were applied as prior constraints, all set with a normal distribution model: (1) the divergence time within *Echinococcus*, with a mean of 6.05 ± 0.605 million years ago (Mya) [18]; (2) the divergence time between *E. canadensis* and *E. ortleppi*, with a mean of 0.58 ± 0.058 Mya [48]; (3) the divergence time between *Taenia asiatica* and *T. saginata*, with a mean of 0.82 ± 0.082 Mya [48]; and (4) the divergence time between *Schistosoma japonicum* and *S. mekongi*, with a mean of 3.86 ± 0.386 Mya [49]. Convergence was assessed using Tracer v1.7.2, with effective sample size (ESS) values greater than 200 used as the threshold for sufficient convergence [50]. The maximum clade credibility tree was then summarized using TreeAnnotator v2.7.7 (part of the BEAST2 package), with the first 25% of the samples discarded as burn-in, and visualized with FigTree v1.4.4 (http://tree.bio.ed.ac.uk/software/figtree/, accessed on 31 October 2025).

A lineage-through-time (LTT) plot for cestodes was generated using the *ape* package in R v4.5.0 [51]. To assess historical population size changes, a Bayesian skyline analysis was performed based on the mitochondrial *cox*1 gene in BEAST v2.7.7 using the coalescent Bayesian skyline as the tree prior and the optimized relaxed molecular clock as the clock model, with a mutation rate of 0.0225 per site per million years applied to the *cox*1 gene [47,52]. The resulting Bayesian skyline plot (BSP) was reconstructed and visualized using Tracer v1.7.2 [50].

## 3. Results

### 3.1. Phylogenetic Inference

**Figure 1:**
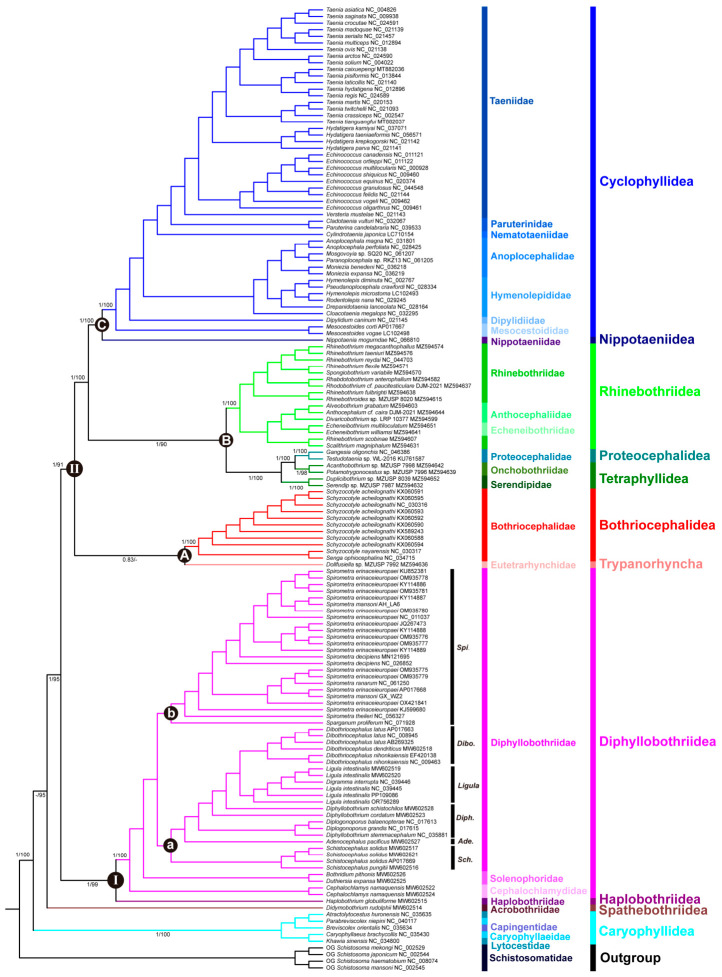
Phylogenetic tree of Cestoda based on 12 protein-coding genes (PCGs). Bayesian posterior probabilities (pp ≥ 0.80) and maximum likelihood bootstrap values (bs ≥ 60) are shown at major nodes. The names of selected genera are abbreviated as follows: *Sch*., *Schistocephalus*; *Ade*., *Adenocephalus*; *Diph*., *Diphyllobothrium*; *Dibo*., *Dibothriocephalus*; *Spi*., *Spirometra*.

Saturation analysis indicated that the dataset comprising the nucleotide sequences of 12 PCGs was suitable for constructing a phylogenetic tree (Supplementary Figure S1). The topological structures of the phylogenetic trees obtained using BI and ML methods were consistent (Figure 1). At the ordinal level, Caryophyllidea diverged earliest, followed by Spathebothriidea. The remaining taxa were divided into two sister clades: Clade I consisted of Haplobothriidea and Diphyllobothriidea. Clade II comprised three groups: Group A diverged first and included Trypanorhyncha and Bothriocephalidea. Group B consisted of Tetraphyllidea, Proteocephalidea, and Rhinebothriidea, with Tetraphyllidea and Proteocephalidea forming a monophyletic clade that is sister to Rhinebothriidea; however, Tetraphyllidea itself was not monophyletic. Group C comprised Nippotaeniidea and Cyclophyllidea. Within Diphyllobothriidea, all three constituent families were monophyletic, with Cephalochlamydidae being sister to a clade consisting of Solenophoridae and Diphyllobothriidae. Within Diphyllobothriidae, two clades were identified. Clade a included five genera: *Schistocephalus*, *Adenocephalus*, *Diphyllobothrium*, *Ligula*, and *Dibothriocephalus*. In this clade, *Schistocephalus* was sister to a monophyletic branch comprising *Adenocephalus*, *Diphyllobothrium*, *Ligula*, and *Dibothriocephalus*. Within this monophyletic branch, *Adenocephalus* diverged first, followed by *Diphyllobothrium*; however, the latter was not monophyletic, with *Ligula* and *Dibothriocephalus* diverging last. Clade b consisted solely of *Spirometra*, suggesting that *Spirometra* represents an independently evolving lineage compared to other diphyllobothriid taxa.

**Figure 2:**
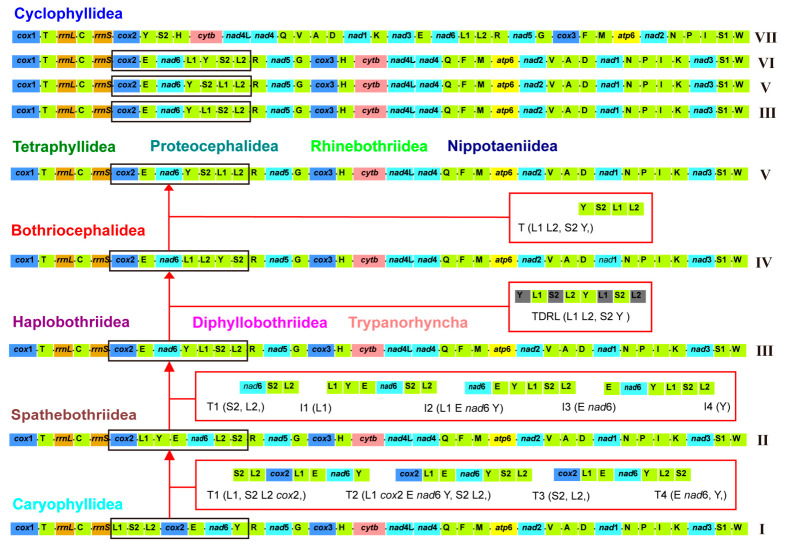
Linear maps of the seven mitochondrial gene arrangement categories in Cestoda, with predicted rearrangement events. Black frames indicate hypervariable regions, arrows indicate direction, and red frames indicate specific events. T, transposition; I, inversion; TDRL, tandem duplication random loss.

The arrangement of the 141 cestode mitogenomes from 11 orders selected in this study (excluding *Testudotaenia* sp. WL-2016, KU761587, due to incomplete tRNA sequences) can be categorized into seven types (I–VII). Types I–VI differed primarily in the order of loci within the variable region L1–S2–L2–*cox*2–E–*nad*6–Y, whereas type VII showed extensive global rearrangement (Figure 2). According to phylogenetic inference, the mitochondrial gene arrangement in Caryophyllidea (the earliest diverging lineage) corresponded to type I. Type II occurred in Spathebothriidea and evolved from type I via four transpositions (T1–T4): T1 exchanged the positions of L1 and the gene block (S2, L2); T2 exchanged the gene blocks (L1, *cox*2, E, *nad*6, Y) and (S2, L2); T3 exchanged S2 and L2; and T4 exchanged the gene block (E, *nad*6) with Y. Type III was shared by Haplobothriidea, Diphyllobothriidea, Trypanorhyncha, and Taeniidae (Cyclophyllidea), evolving from type II through one transposition (T1) and four inversions (I1–I4): T1 exchanged S2 and L2; I1 reversed L1 without changing its order; I2 reversed the entire gene block (L1, E, *nad*6, Y); I3 reversed the gene block (E, *nad*6); and I4 reversed the single gene Y. Type IV was unique to Bothriocephalidea and evolved from type III through a tandem duplication random loss (TDRL) event, where L1 and L2 were retained in the first copy (type III), while S2 and Y were retained in the last copy (type IV). Type V was present in Tetraphyllidea, Proteocephalidea, Rhinebothriidea, Nippotaeniidea, and five families within Cyclophyllidea: Paruterinidae, Anoplocephalidae (genera *Anoplocephala*, *Mosgovoyia*, and *Moniezia*), Hymenolepididae, Dipylidiidae, and Mesocestoididae. The transition from type IV to type V involved one transposition event that exchanged the gene blocks (L1, L2) and (S2, Y). Type VI was unique to *Paranoplocephala* within Anoplocephalidae (Cyclophyllidea). The evolution from either type III or type V to type VI involved only a single transposition event: in the former case, the L1 and Y genes exchanged positions, while in the latter, the gene block (Y, S2) exchanged positions with the L1 gene. Type VII, exclusive to Nematotaeniidae (Cyclophyllidea), displayed extensive reorganization and represented the most distinct arrangement among cestodes.

### 3.2. Species Identification of Spirometra

**Figure 3:**
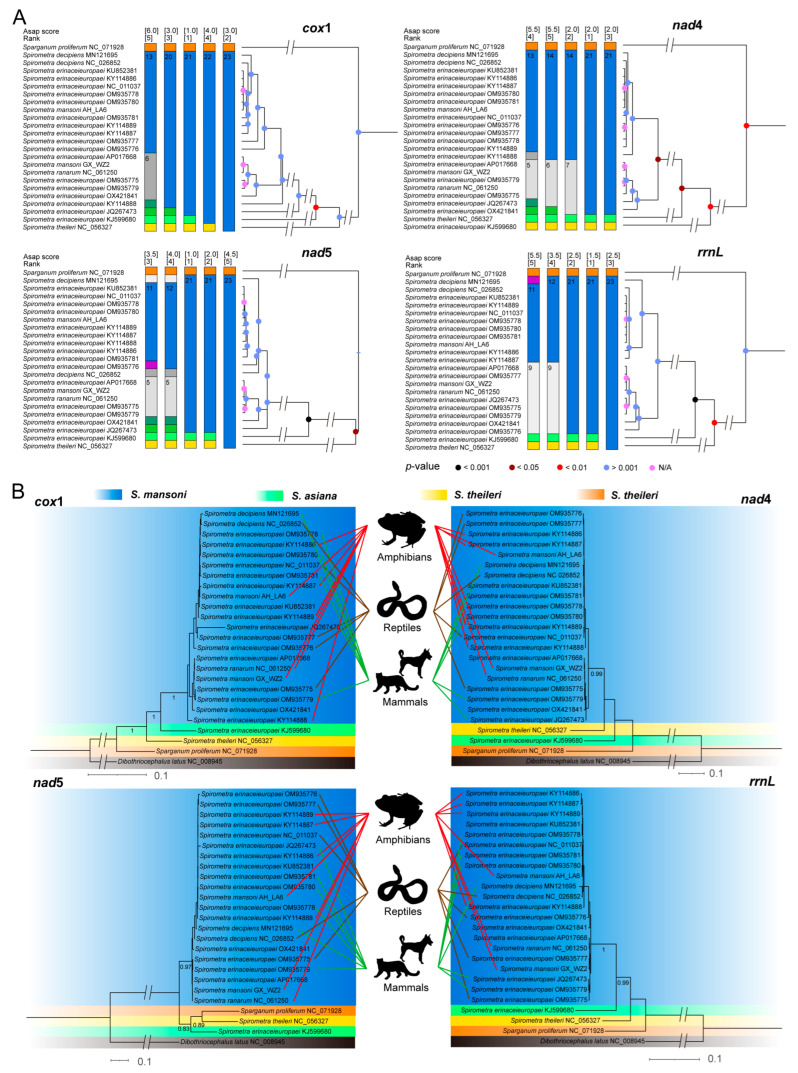
Species delineation in *Spirometra* based on *cox*1, *nad*4, *nad*5, and *rrnL*. (**A**) ASAP analyses. The numbers in the bars represent the number of individuals identified as the same species. (**B**) Bayesian inference analyses.

**Figure 4:**
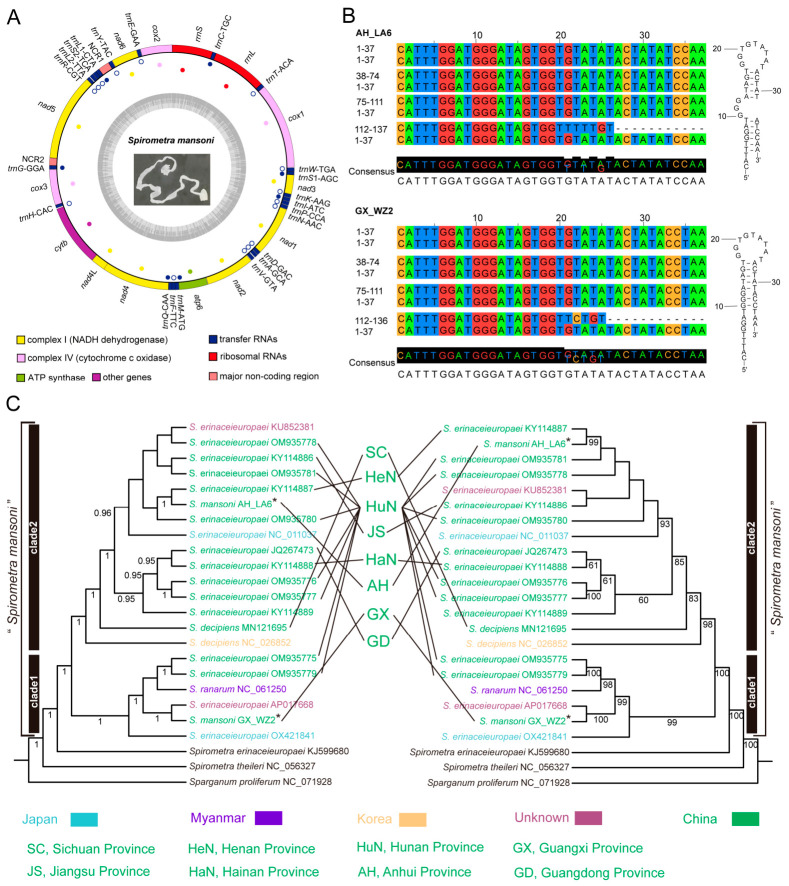
Mitochondrial genome and phylogeny of *Spirometra mansoni*. (**A**) Circular mitogenome map, with dots representing nucleotide similarity between the AH_LA6 and GX_WZ2 isolates (solid = variable, hollow = identical). (**B**) Tandem repeats and predicted secondary structures in the NCR2 of both isolates. (**C**) Phylogeny of the *Spirometra* clade. This subtree was extracted from the broader phylogenetic framework shown in Figure 1. Bayesian posterior probabilities (pp ≥ 0.80) and maximum likelihood bootstrap values (bs ≥ 60) are shown at major nodes. Asterisks (*) indicate newly sequenced samples.

To determine the number of valid *Spirometra* species, we first assessed the genetic diversity of 24 available mitogenomic sequences, identifying 3079 polymorphic sites with an overall *π* of 0.042. Sliding window analysis revealed the greatest diversity in *cox*1 (0.074), *nad*4 (0.078), *nad*5 (0.084), and *rrnL* (0.085), suggesting that these regions are suitable for species-level discrimination (Supplementary Figure S2). Consequently, these four genes were selected for ASAP analysis. The ASAP results supported an optimal partition into four species across all four genes. Under this partition, *Sparganum proliferum* (NC_071928), *S. theileri* (NC_056327), and *S. erinaceieuropaei* (KJ599680) were each delimited as distinct species, while the remaining 21 sequences formed a fourth group. Notably, these 21 sequences were inconsistently annotated under four names: *S. decipiens*, *S. erinaceieuropaei*, *S. mansoni*, and *S. ranarum* (Figure 3A). Phylogenetic analysis based on *cox*1, *nad*4, *nad*5, and *rrnL* consistently divided *Spirometra* into four clades: *S. proliferum*, *S. theileri*, and *S. erinaceieuropaei* (KJ599680), each forming a distinct clade, corresponding to the revised *Spirometra* sp. 2, *S. theileri*, and *S. asiana*, respectively, while the remaining sequences clustered into a single intermixed clade, corresponding to the revised *S. mansoni* (Figure 3B). This delineation was consistent with the ASAP species delimitation results. Additionally, the newly sequenced strains AH_LA6 and GX_WZ2, representing two genetic lineages of *S. mansoni*, differed by only about 2% in sequence across 12 PCGs, 7 tRNAs (*trnC*-TGC, *trnL*1-CTA, *trnG*-GGA, *trnQ*-CAA, *trnM*-ATG, *trnI*-ATC, and *trnS*1-AGC), 2 rRNAs, and 2 mNCRs (NCR1 and NCR2) (Figure 4A, Supplementary Tables S4 and S5). Specifically, among the seven differential tRNAs, six exhibited single-base substitutions, while *trnL*1 showed a single-base insertion in its secondary structure (Supplementary Figures S3 and S4). In NCR2, within a 37 bp tandem repeat (TR) sequence, a T base shifted from position 33 in AH_LA6 to position 35 in GX_WZ2 (Figure 4B). BI and ML analyses of the 21 *S. mansoni* sequences consistently supported two well-supported clades (Clade 1 and Clade 2; pp = 1.00; bs = 100) (Figure 4C). Clade 1 contained isolates from Japan, China, and Myanmar, while Clade 2 included those from Japan, China, and South Korea. Within China, the isolates were distributed across both clades: three from Guangxi and Hunan belonged to Clade 1, and twelve from Sichuan, Henan, Jiangsu, Hainan, Anhui, Guangdong, and Hunan belonged to Clade 2. These results confirmed the presence of two *S. mansoni* genotypes in China.

### 3.3. Ancestral Character Reconstruction

To investigate the evolutionary history of cestodes, ancestral state reconstruction was conducted for four traits: host habitat, life-cycle host number, second intermediate host type, and definitive host type. Notably, Spathebothriidea, Haplobothriidea, Trypanorhyncha, and Nippotaeniidea were each represented by a single species, precluding ancestral inference for these groups. Tetraphyllidea and Proteocephalidea were analysed together because they form a monophyletic unit and Tetraphyllidea is paraphyletic. Within the Diphyllobothriidae, *Adenocephalus* was not included in the analysis as it is a monospecific genus; within *Diphyllobothrium*, due to the taxonomic uncertainty among pinniped parasites, the analysis focused on the monophyletic cetacean-associated species.

**Figure 5:**
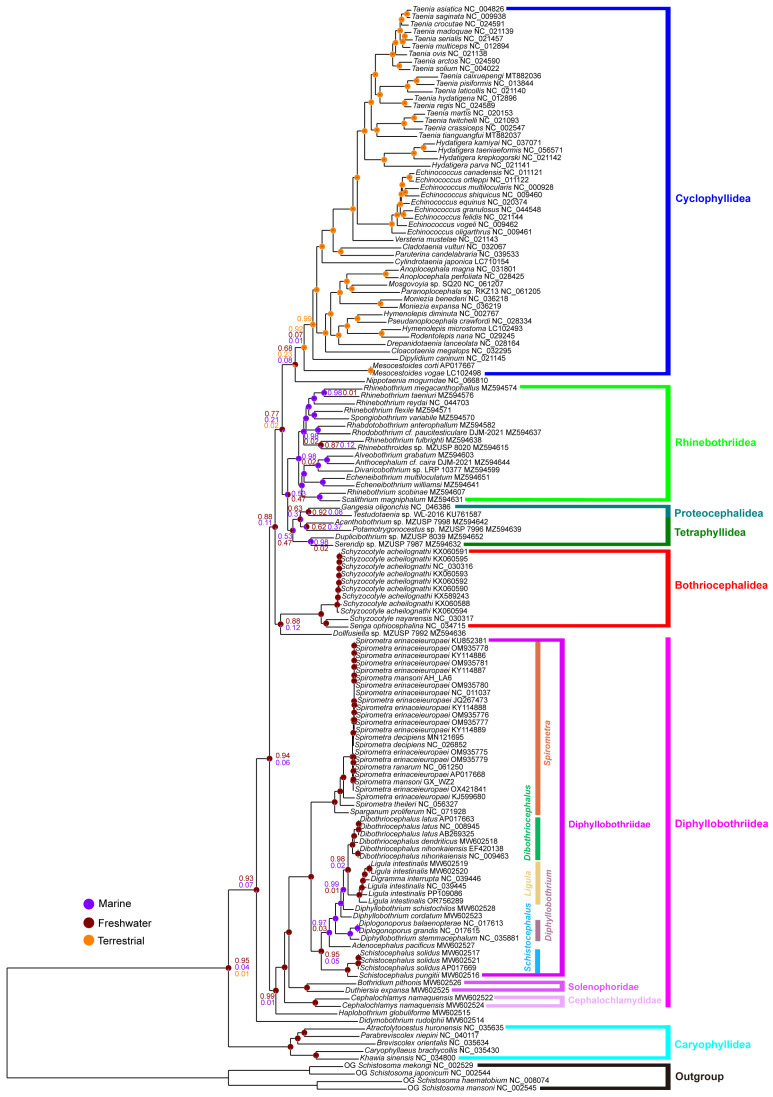
Ancestral reconstruction of host habitats in cestodes. Node colours represent the probability of state occurrence, with numerical values shown only for uncertain nodes (unlabelled nodes have a probability of 1.0).

Reconstruction based on host habitat suggested that Cestoda most likely originated in freshwater environments (0.95 for freshwater vs. 0.04 for marine and 0.01 for terrestrial) (Figure 5). Caryophyllidea, the earliest diverging lineage, was inferred to be derived from freshwater. The remaining groups formed a clade that was also likely of freshwater origin (0.93 for freshwater vs. 0.07 for marine). This clade split into two main lineages: one primarily consisting of Diphyllobothriidea, which was also inferred to be of freshwater origin. The three families within Diphyllobothriidea were all reconstructed as being of freshwater origin. Within Diphyllobothriidae, *Schistocephalus*, *Dibothriocephalus*, *Ligula*, and *Spirometra* originated in freshwater, whereas *Diphyllobothrium* (cetacean parasites) arose in marine habitats. The other lineages included primarily Bothriocephalidea, Proteocephalidea + Tetraphyllidea, Rhinebothriidea, and Cyclophyllidea. Among these, Bothriocephalidea was clearly of freshwater origin. Tetraphyllidea + Proteocephalidea had nearly equal probability for marine and freshwater origin (0.53 for marine vs. 0.47 for freshwater), whereas Rhinebothriidea was strongly supported as being of marine origin (0.98 for marine vs. 0.02 for freshwater). In contrast, Cyclophyllidea was strongly inferred to be terrestrial in origin (0.92 for terrestrial vs. 0.07 for freshwater and 0.01 for marine).

Ancestral state reconstruction based on life-cycle host number indicated that most cestode groups exhibited a two-host pattern. A three-host pattern occurred primarily in Diphyllobothriidae and Solenophoridae (Diphyllobothriidea), as well as in Mesocestoididae (Cyclophyllidea) (Supplementary Figure S5). For taxa with three-host cycles, ancestral analysis of the second intermediate host suggested that the ancestor of Diphyllobothriidae likely used fish (0.55 for fish vs. 0.24 for tetrapods, 0.18 for absent, and 0.04 for unknown), whereas *Spirometra* later shifted to amphibians. The ancestral second intermediate host for Solenophoridae remained uncertain (0.97 for unknown vs. 0.03 for absent), and that of Mesocestoididae was inferred to be rodents (Supplementary Figure S6).

**Figure 6:**
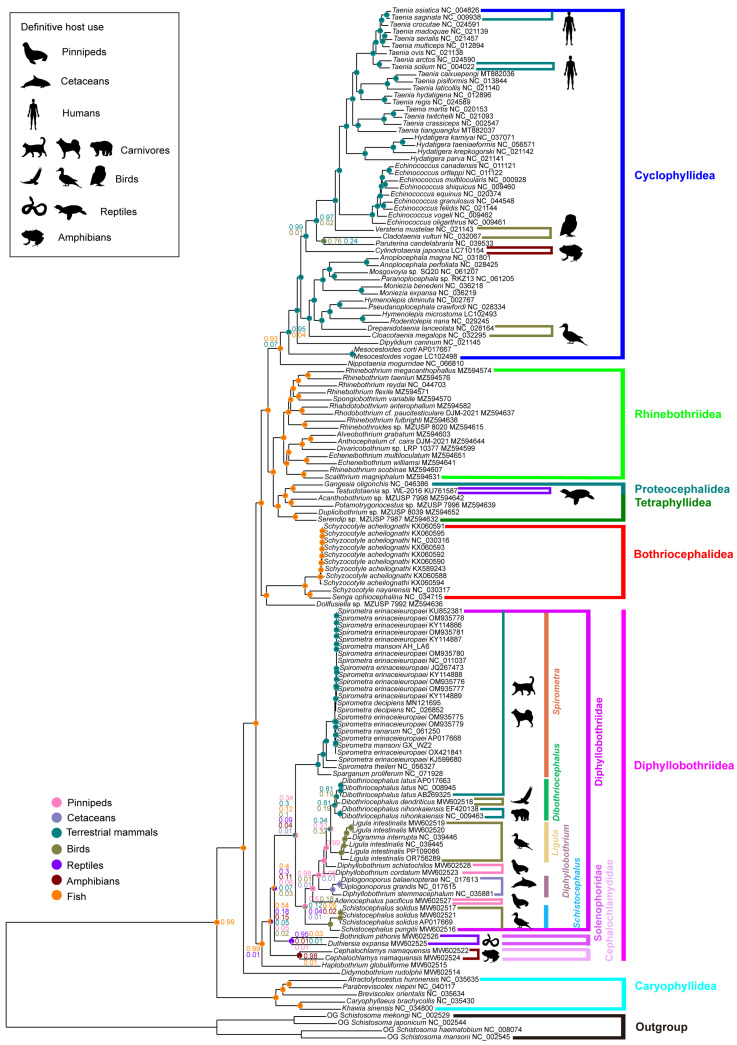
Ancestral reconstruction of definitive host types in cestodes. Node colours indicate the probability of state occurrence, with numerical values shown only for uncertain nodes (unlabelled nodes have a probability of 1.0). For Diphyllobothriidea, all the definitive hosts are labelled. For other taxa, only hosts that differ from those with typical group characteristics are labelled. Definitive hosts of Cyclophyllidea are also labelled for species that are exclusively parasitic in humans.

Reconstruction based on the definitive host type indicated that Cestoda likely originated with fish as the definitive host (0.99 for fish) (Figure 6). Caryophyllidea, the earliest diverging lineage, also had a fish-host ancestor. The remaining groups formed a clade whose ancestor similarly used fish. This clade split into two main lineages. In one lineage, the ancestor of Diphyllobothriidea likely retained fish as the definitive host (0.54 for fish vs. 0.18 for reptiles, 0.15 for amphibians, 0.05 for terrestrial mammals, 0.05 for pinnipeds, and 0.02 for birds). Within this order, Cephalochlamydidae later shifted to amphibians (0.98 for amphibians vs. 0.01 for fish), Solenophoridae shifted to reptiles (0.95 for reptiles vs. 0.03 for fish, 0.01 for amphibians, 0.01 for terrestrial mammals, and 0.01 for pinnipeds), and Diphyllobothriidae showed a complex ancestral state (0.34 for pinnipeds vs. 0.3 for terrestrial mammals, 0.12 for fish, 0.1 for birds, 0.09 for reptiles, 0.04 for amphibians, and 0.01 for cetaceans). In Diphyllobothriidae, *Spirometra* ancestrally used terrestrial mammals, whereas the ancestor of the remaining genera (*Schistocephalus*, *Ligula*, *Diphyllobothrium*, *Dibothriocephalus*) likely used pinnipeds (0.6 for pinnipeds vs. 0.18 for birds, 0.12 for terrestrial mammals, 0.05 for fish, 0.04 for reptiles, 0.02 for amphibians, and 0.01 for cetaceans). Subsequent diversification led to further host shifts: the ancestors of *Schistocephalus* and *Ligula* utilized birds, the ancestor of *Diphyllobothrium* (restricted to the monophyletic lineage) utilized cetaceans, and the ancestor of *Dibothriocephalus* likely utilized terrestrial mammals (0.81 for terrestrial mammals vs. 0.19 for birds). Notably, within Diphyllobothriidae, only the taxonomically uncertain *Diphyllobothrium cordatum* and *Diphyllobothrium schistochilos* retained pinnipeds as definitive hosts. In the other lineage, the ancestors of Bothriocephalidea, Tetraphyllidea + Proteocephalidea, and Rhinebothriidea all used fish as definitive hosts. In contrast, the ancestor of Cyclophyllidea shifted to terrestrial mammals (0.95 for terrestrial mammals vs. 0.04 for fish). Within Cyclophyllidea, some species, such as *T. asiatica*, *T. saginata*, and *T. solium*, further evolved to utilize humans as their sole definitive host.

### 3.4. Molecular Clock Analysis

**Figure 7:**
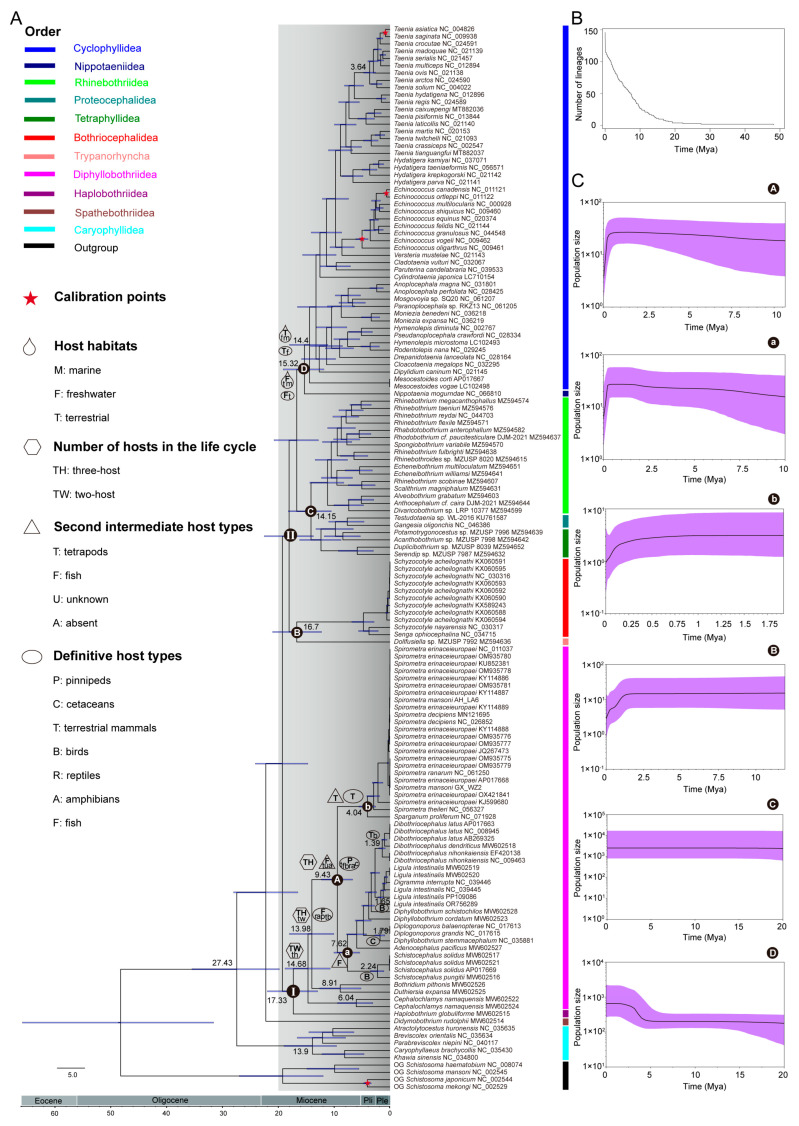
Divergence time estimation in tapeworms. (**A**) BEAST analysis based on 12 PCGs and 2 rRNAs. Node numbers represent the ages of the major lineage. Ancestral states are abbreviated, with bold uppercase indicating the most likely state. (**B**) Lineage-through-time plot corresponding to the BEAST chronogram. (**C**) Bayesian skyline plots for the six major lineages based on *cox*1, showing median estimates (solid line) and 95% highest posterior probability density (HPD) intervals (blue shading).

To estimate divergence times among cestode groups, we constructed a phylogenetic consensus tree using BEAST analysis (Figure 7A). The results indicated that the diversification of Cestoda began in the mid-to-late Oligocene (27.43 Mya). Caryophyllidea was the first to diverge, with further diversification occurring at 13.9 Mya (95% HPD: 9.56–18.99). The remaining taxa formed two main clades: Clade I included Haplobothriidea and Diphyllobothriidea, which shared a common ancestor dating to 17.33 Mya (95% HPD: 12.94–21.98). Within Diphyllobothriidea, Cephalochlamydidae diverged first, followed by Solenophoridae and then Diphyllobothriidae (Subclade A), with their subsequent diversification occurring at 6.04 Mya (95% HPD: 3.07–9.42), 8.91 Mya (95% HPD: 5.19–12.62), and 9.43 Mya (95% HPD: 6.69–12.29), respectively. Subclade A consisted of two sister groups. Group a originated at 7.62 Mya (95% HPD: 5.42–10.06), with later diversification events at 2.24 Mya (95% HPD: 1.08–3.53) for *Schistocephalus*, 1.79 Mya (95% HPD: 0.91–2.76) for *Diphyllobothrium* (cetacean parasites), 1.65 Mya (95% HPD: 1.11–2.19) for *Ligula*, and 1.39 Mya (95% HPD: 0.84–2.03) for *Dibothriocephalus*. Group b, which contained only *Spirometra*, began diversifying at 4.04 Mya (95% HPD: 2.57–5.56). Clade II contained three subclades: Subclade B (Trypanorhyncha and Bothriocephalidea), which diverged from 16.7 Mya (95% HPD: 12.25–21.03); Subclade C (Tetraphyllidea + Proteocephalidea and Rhinebothriidea), which originated at 14.15 Mya (95% HPD: 10.51–18.21); and Subclade D (Nippotaeniidea and Cyclophyllidea), which originated at 15.32 Mya (95% HPD: 11.81–19.13). Cyclophyllidea, the major group within Subclade D, further diversified from 14.4 Mya (95% HPD: 11.12–18.01).

We also conducted diversification rate analyses to investigate the impact of habitat and host shifts on tapeworm evolution. The lineage-through-time (LTT) plot indicated that cestodes entered a phase of sustained rapid radiation from the early Miocene (20 Mya) onwards, with a further marked acceleration from the Pleistocene (2 Mya) to the Holocene (Figure 7B). The integration of the ancestral state reconstruction and divergence time estimation revealed key evolutionary transitions during this radiation. First, a shift from aquatic to terrestrial host habitats began in the early Miocene (15.32 Mya), corresponding to the divergence between Nippotaeniidea and Cyclophyllidea. By the mid-Miocene (14.4 Mya), Cyclophyllidea lineages had largely transitioned to terrestrial hosts. Second, with respect to a life-cycle strategy, a transition from two- to three-host cycles occurred around the mid-Miocene (14.68 Mya), aligning with the divergence between Cephalochlamydidae and Diphyllobothriidae+Solenophoridae. By the late Miocene (9.43 Mya), Diphyllobothriidae had fully adopted a three-host cycle. Concurrently, second intermediate hosts diversified. By the end of the Miocene (7.62 Mya), lineages in Group a utilized fish, whereas by the early Pliocene (4.04 Mya), lineages in Group b utilized tetrapods, mainly amphibians. Finally, shifts in definitive host preference were observed. In Diphyllobothriidea, the ancestral host shifted from fish to pinnipeds in the mid-Miocene (13.98 Mya) and later to terrestrial mammals in the late Miocene (9.43 Mya), corresponding to the divergence between Groups a and b. After this, Group a radiated extensively during the Pleistocene (1.39–2.24 Mya), adapting to various hosts, including cetaceans, birds, and terrestrial mammals. In Cyclophyllidea, the shift from fish to terrestrial mammals occurred earlier, in the early Miocene (15.32 Mya), which coincided with the habitat transition of their hosts. Notably, human-specialized tapeworms within Cyclophyllidea likely began specializing in humans in the early Pliocene (3.64 Mya).

Additionally, we constructed Bayesian skyline plots (BSPs) for the major monophyletic subclades (A–D) to analyse the effects of habitat and host evolution on tapeworm population dynamics (Figure 7C). The results revealed distinct historical trajectories of effective population size among these lineages. Subclade A declined sharply after a long period of slow expansion, a pattern also reflected in its internal Group a, while Group b initially contracted slowly but then decreased rapidly before a slight recovery. Subclade B remained stable for an extended period before undergoing a rapid decline. Subclade C remained largely stable throughout, whereas Subclade D expanded rapidly after a stable phase and then stabilized again. These findings suggest that while habitat and host shifts may have had limited short-term effects on population size, long-term interactions between tapeworms, their host lineages, and environmental factors likely played a key role in shaping their demographic histories.

## 4. Discussion

The taxonomy of diphyllobothriid tapeworms has long been problematic, and the status of several pathogenic taxa remains controversial. To address these issues, we reconstructed the phylogenetic relationships of 11 cestode orders based on mitochondrial genomes, evaluated the higher-level taxonomic framework, resolved internal relationships within Diphyllobothriidae, delimited valid species in *Spirometra*, explored the differences between the two genetic lineages of *S. mansoni*, and conducted ancestral character reconstruction and divergence time estimation. Although these results provide new insights into the evolution of this group, several limitations should be noted. Mitochondrial genes are subject to compositional bias, rate heterogeneity, and substitution saturation, which may lead to topological errors or biased divergence time estimates, particularly in groups lacking robust fossil calibrations [53,54]. In addition, taxonomic sampling in this study is uneven, and sequences for some *Spirometra* taxa are also missing, largely due to the limited availability of complete mitochondrial genomes in public databases. Such uneven sampling may lead to long-branch attraction or bias in ancestral state reconstruction, potentially affecting inferences at some deep nodes [55]. Future studies should further sample rare lineages and incorporate nuclear or genomic data for further validation.

Phylogenetic analysis based on mitochondrial genome data showed that Caryophyllidea is the earliest diverging cestode lineage. This result differs from earlier studies based on nuclear rDNA (small subunit (SSU) and large subunit (LSU) D1-D3 regions), which identified Spathebothriidea as the most basal lineage, a discrepancy that may arise from the use of different molecular markers [10]. Diphyllobothriidea and Haplobothriidea formed a well-supported clade (Clade I). In contrast, Bothriocephalidea clustered with Trypanorhyncha as Group A (within Clade II). These findings support the view that Diphyllobothriidea represents a relatively basal lineage, while Bothriocephalidea is closer to derived “tetrafossate” tapeworms such as Proteocephalidea and Cyclophyllidea [11,13]. However, constrained by the limited number of taxa, the sister group relationship between Bothriocephalidea and Trypanorhyncha remains to be further confirmed, contrary to previous findings that identified Trypanorhyncha and Diphyllidea as sister groups [10]. Rhinebothriidea first grouped with Tetraphyllidea + Proteocephalidea to form Group B, which then formed a sister group with the clade comprising Cyclophyllidea and Nippotaeniidea. This topology differs from previous findings, indicating that Rhinebothriidea directly forms a sister group to the clade containing the above three groups, a difference that may be related to the number of representative species and the molecular markers used [56]. Within Diphyllobothriidea, Cephalochlamydidae diverged first, with Solenophoridae and Diphyllobothriidae forming sister groups, consistent with recent studies [2,7]. The six genera within Diphyllobothriidae were divided into two independent clades, which is also consistent with recent findings [2]. Within Clade a, the earliest diverging genus, *Schistocephalus*, uses waterbirds or other piscivorous birds as definitive hosts, whereas the last diverging sister group, *Ligula* and *Dibothriocephalus*, shows divergent host preferences: *Ligula* still parasitizes waterbirds or piscivorous birds, whereas *Dibothriocephalus* exhibits high definitive host diversity, including cats, dogs, piscivorous birds, and bears [1]. This difference in host utilization suggests two independent evolutionary events in definitive host selection. Notably, the taxonomic relationship between *Dibothriocephalus* and *Diphyllobothrium* has long been confused [7]. This study confirmed the monophyly of *Dibothriocephalus*, supporting its validity at the generic level. Although *Diphyllobothrium* has undergone several revisions, it remains nonmonophyletic, and the phylogenetic positions of some species (*Diphyllobothrium cordatum* and *Diphyllobothrium schistochilos*) are still unresolved [1,57,58]. The closer phylogenetic relationship between *Dibothriocephalus* and *Ligula* is also consistent with ecological evidence, namely that *Dibothriocephalus* and *Ligula* primarily parasitize freshwater or terrestrial animal hosts, whereas *Diphyllobothrium* mainly parasitizes marine mammal hosts [7].

Mitogenomic sequences are highly conserved, but gene rearrangements occur across taxa and can indicate evolutionary relationships, as closely related groups often share identical gene orders [59]. In this study, analysis of gene arrangements in 141 cestode mitogenomes from 11 orders revealed seven types (I–VII) that corresponded to lineage divergence. Three new types were identified compared with those in previous reports, including one in Spathebothriidea (II) and two in Cyclophyllidea (VI and VII) [13]. Only types III and V were shared across multiple orders, indicating greater stability and wider distribution. Interestingly, mitochondrial gene arrangement in cestodes appears to be closely linked to proglottid evolution. In lineages with poorly differentiated scoleces, gene order evolved from type I (Caryophyllidea) to type II (Spathebothriidea) through four transposition events, corresponding to the shift from a monozoic body to incomplete segmentation. The transition from type II to type III, involving one transposition and four reversals, coincided with the development of clear segmentation and scolex differentiation into tentaculate forms (Haplobothriidea) and bothriate forms (Diphyllobothriidea and Trypanorhyncha). The shift from type III to type IV (Bothriocephalidea) required only one tandem duplication random loss event, whereas reverting to type III would require two transpositions, suggesting that type III is likely ancestral to type IV [13]. This finding supports the origin of Diphyllobothriidea being earlier than that of Bothriocephalidea despite their shared scolex type. One transposition from type IV to type V aligned with the shift from bothriate scoleces to those with bothria, bothridia or suckers (Tetraphyllidea, Proteocephalidea, Rhinebothriidea, and Nippotaeniidea), implying that type V represents a morphologically diversified form. Notably, Cyclophyllidea, which possesses suckered scoleces, displayed four gene arrangement types (III, V, VI, and VII), indicating more complex mitogenomic evolution and suggesting the diversification of scolex types at the type III stage. Within Cyclophyllidea, types III, V, and VI differ by only one translocation, whereas type VII shows extensive reorganization, suggesting the following two evolutionary pathways: gradual local adjustments and major rearrangement possibly driven by structural variants. Overall, increasing scolex complexity generally appears to align with phylogenetic progression, and more divergent lineages tend to exhibit relatively complex gene arrangements; however, this pattern remains to be formally tested with expanded taxon sampling and suitable comparative methods.

ASAP analysis based on four genes showed that among the top five partitioning schemes, the optimal partition corresponding to the lowest ASAP score was consistently 4, indicating that dividing all 24 *Spirometra* sequences into four species is the most appropriate. This delimitation was further supported by phylogenetic reconstruction, particularly in that the 21 previously inconsistently named sequences were all clustered within the revised *S. mansoni* clade (all Bayesian posterior probabilities > 0.95), which is consistent with recent taxonomic studies [15,20,21,60]. Therefore, we revised the names of these four valid species following the latest *Spirometra* classification system. We selected four mitochondrial genes with relatively high variability for species delimitation in *Spirometra*, mainly because genetic divergence among species in this genus is relatively low, and current taxonomic revisions still rely primarily on *cox*1 as the reference marker [15,20]. However, since most of the sequences used in this study were derived from public databases, we were unable to obtain corresponding nuclear genes for verification, and mitochondrial markers represent only maternal lineages, which cannot capture nuclear gene flow or hybridization events [61]. Therefore, this delimitation still requires further validation with nuclear markers and morphological evidence. Differences in PCGs between the two geographical strains of *S. mansoni* led to variations in amino acid usage and codon preference, which were likely influenced by asymmetric base composition (AT- and GC-skews) associated with strand displacement replication [62]. Structural variations in seven tRNAs, involving base substitutions and insertions, can affect tRNA stability and mitochondrial translation efficiency [63]. In the NCR2 region, although the repeat unit count and stem–loop-forming ability were conserved, single-base positional variations were observed between isolates, resembling patterns reported in related species [12]. In contrast, more distantly related Caryophyllidea species exhibited tandem repeats in both NCR1 and NCR2, with NCR2 containing up to 20 repeats [13]. Phylogenetic analysis of the 12 PCGs confirmed that *S. mansoni* (primarily comprising Asian isolates) formed two distinct clades, a result consistent with findings based on four genes. Chinese and Japanese isolates were distributed across both clades, indicating that these lineages were not geographically isolated and likely coexisted across East Asia. Notably, while both clades were present in China, Clade 1 appeared limited to Guangxi and Hunan, whereas Clade 2 was widespread nationwide. This distinct distribution pattern requires further study.

Ancestral reconstruction indicated a freshwater fish origin for Cestoda, which is consistent with its phylogeny: the earliest diverging extant eucestodes are Caryophyllidea, which parasitize freshwater fish [64]. For Diphyllobothriidea, our results support a freshwater origin, with fish as the most likely ancestral definitive host (0.54 for fish), in contrast with an earlier inference of reptile origin [2]. Within Diphyllobothriidea, the three families showed distinct ancestral host preferences: Cephalochlamydidae likely originated in amphibians, Solenophoridae in reptiles, and Diphyllobothriidae remained uncertain between pinnipeds and terrestrial mammals (0.34 vs. 0.30, respectively). However, Fraija-Fernández et al. proposed carnivores as the more likely ancestral definitive host of Diphyllobothriidae [2]. This study was the first to elucidate the evolution of life cycles across these families. The results revealed that Cephalochlamydidae ancestrally employed a two-host cycle, whereas Solenophoridae and Diphyllobothriidae independently evolved three-host cycles. Within Diphyllobothriidae, ancestral state reconstruction indicated that *Schistocephalus*, *Dibothriocephalus*, *Ligula*, and *Spirometra* originated in freshwater environments, whereas *Diphyllobothrium* (cetacean parasites) arose in marine habitats. With respect to second intermediate hosts, analysis suggested that *Spirometra* likely ancestrally utilized amphibians, whereas fish served as the probable ancestral host for the other four genera. However, due to unavailable mitogenomic data, recently reported *Spirometra* species that parasitize fish could not be included [60]. With respect to definitive hosts, the ancestor of *Spirometra* parasitized terrestrial mammals, *Schistocephalus* and *Ligula* ancestors parasitized birds, and the ancestor of *Diphyllobothrium* (restricted to the monophyletic lineage) parasitized cetaceans, whereas *Dibothriocephalus* most likely originated in terrestrial mammals (0.81 for terrestrial mammals). These distinct host-use and habitat patterns support the taxonomic revision of *Diphyllobothrium* by Waeschenbach et al., who emphasized ecological and host differences. Ancestral inference revealed that Cyclophyllidea more likely originated in terrestrial environments (0.92 for terrestrial) and that its ancestral definitive host was most likely terrestrial mammals (0.95 for terrestrial mammals) [7]. Some *Taenia* species even exclusively parasitize humans. This raises the question of how tapeworms transitioned from initially infecting freshwater fish to adapting to terrestrial mammals. We propose the following hypotheses: First, host switching facilitated the shift from freshwater to land. Amphibians could have carried parasites to land–water interfaces, where omnivorous reptiles and birds consuming amphibian prey introduced larvae into terrestrial food chains [1]. Furthermore, through their broad diet and movement, waterbirds and migratory birds may have further transported tapeworms inland, enabling full terrestrial adaptation [1]. Second, the emergence of Cyclophyllidea marked complete adaptation to terrestrial hosts, exploiting mammals as a new parasitic niche. To survive in arid settings and enhance transmission, tapeworms internalized egg development within the intestine of the definitive host. Their reproductive strategy shifted from releasing individual eggs to releasing gravid proglottids, where the uterus substitutes for the protective role of the eggshell, ensuring egg viability until ingestion by intermediate hosts [8,65]. Humans likely entered this cycle through hunting, livestock domestication, or the consumption of undercooked meat [66]. In addition to the major evolutionary transition from aquatic to terrestrial environments, some tapeworm lineages have independently transitioned from freshwater to marine environments. This shift may have been mediated by euryhaline or migratory fish hosts, which move between salinity regimes, providing a pathway for parasites to adapt to marine conditions. Notably, intestinal tapeworm communities in marine sharks show species richness and infection patterns resembling those in freshwater fish, suggesting a freshwater origin for these parasites [67].

The evolutionary radiation of cestodes has been closely linked to global climate change and host turnover since the Cenozoic [66]. Molecular clock analyses revealed major radiation beginning in the mid-to-late Oligocene (27.43 Mya), which notably accelerated by the mid-Miocene. This trend likely corresponded to the rapid diversification of mammals and birds during this period in response to climate change [68,69]. The time tree revealed that during this period, two orders in Clade I (Haplobothriidea and Diphyllobothriidea) diverged, along with seven orders across three subclades within Clade II. This pattern suggests that host diversity is a key driver of cestode differentiation. Within Diphyllobothriidea, the radiation of three families corresponded with their host groups and life cycles. Diphyllobothriidae radiated earliest in the late Miocene, aligning with the peak diversification of mammals and birds, which facilitated their complex life cycles involving vertebrate intermediate hosts [1]. In contrast, Cephalochlamydidae, with a narrow host range (frogs) and a simple one-host cycle, radiated last, extending into the late Pliocene, possibly reflecting host-limited diversification [7]. Solenophoridae radiated between these two families and may represent a transitional evolutionary stage, with a life cycle that potentially involves a second intermediate host [2]. Within Diphyllobothriidae, genus-level radiations showed a stepwise pattern: *Spirometra* radiated in the Pliocene, possibly linked to grassland expansion, whereas other genera radiated primarily in the Pleistocene, a period of human evolution and increased global activity that may have influenced parasite transmission [70,71]. Cyclophyllidea became a major group after its differentiation in the middle Miocene. During this time, its definitive hosts shifted from fish to terrestrial mammals, matching the rapid diversification of mammals and the ecological transition from aquatic to terrestrial environments [69]. Obligate human parasites had already emerged by the Early Pliocene. Their origin slightly predates the putative host-switching event, which likely occurred during the Pliocene–Pleistocene transition. During this period, climatic and ecological changes facilitated the shared utilization of bovid intermediate hosts, potentially driving the host shift of parasites from carnivores to early humans [72]. Fossil evidence indicates that humans in the early Pleistocene were hunting large ungulates or scavenging, suggesting that the origin of human tapeworms is older and more complex than previously thought and not solely linked to animal domestication in the Holocene [73]. The inferred Pliocene origin for human-specific *Taenia* spp. in this study is earlier than the Pliocene–Pleistocene transition, possibly because these parasites do not form a monophyletic clade, and their timing was estimated based on the earliest clade they share with other species. The exact origins and host-switching mechanisms of *T. solium*, *T. asiatica*, and *T. saginata* require further research. Furthermore, the molecular clock calibration in this study was primarily based on well-studied genera such as *Echinococcus*, *Taenia*, and *Schistosoma*, with the calibration points within these genera representing relatively recent divergence events, which may increase uncertainty when extrapolating to deeper nodes. This limitation is difficult to fully avoid in the present study, as reliable fossil records for Cestoda are extremely limited. We therefore referred to calibration points previously used in the literature and applied a partitioned model with a relaxed molecular clock to better accommodate rate variation among different gene loci. Under this framework, the relative ages of major deep nodes are comparable to those reported in earlier studies, although further constraints from additional fossil discoveries or genomic-level secondary calibrations are still needed [18].

## 5. Conclusions

Firstly, the phylogenetic analysis supported Caryophyllidea as the earliest diverging lineage and confirmed that Diphyllobothriidea and Bothriocephalidea are distinct clades. Additionally, seven types of mitochondrial gene arrangements were identified, three of which were newly discovered, and variations in gene arrangement were found to correlate with the evolution of proglottid morphology in tapeworms. Secondly, species validation indicated that the available *Spirometra* mitogenomes represent only four valid species, and the representative sequences of the two *S. mansoni* lineages differed in 12 PCGs, 7 tRNAs, 2 rRNAs, and 2 mNCRs. Thirdly, ancestral state reconstruction indicated that cestodes likely originated in freshwater fish, with the transition and adaptation to terrestrial hosts representing the main pathway of diversification, whereas colonization of marine environments was an independent evolutionary event in a few lineages. Finally, molecular clock divergence time estimation suggested that cestodes began to radiate from the middle to late Oligocene, with the radiation of most genera in Diphyllobothriidae concentrated in the Pleistocene, while the radiation of the *Spirometra* occurred during the Pliocene. In summary, this study provides a framework for understanding tapeworm origins, diversification mechanisms, and the evolutionary processes shaping pathogenic lineages.

## Data Availability

The original contributions presented in this study are included in the article and the Supplementary Materials. Further inquiries can be directed to the corresponding author.

## Supplementary Materials

The following supporting information can be downloaded at: https://www.mdpi.com/article/10.3390/ani16132084/s1, Table S1: Mitochondrial genome sequences of the cestodes used in this study. Asterisks (*) indicate newly obtained sequences. Table S2: Best models selected under the Bayesian information criterion (BIC) for each subset partition in this study. Table S3: Classification of cestodes based on four traits. 1 indicates that the trait is present, and 0 indicates that the trait is absent. Habitat: T (terrestrial), F (freshwater), M (marine). Number of hosts: O (one), Tw (two), Th (three). Second intermediate host: A (absent), U (unknown), F (fish), T (tetrapods). Definitive host: F (fish), A (amphibians), R (reptiles), B (birds), T (terrestrial mammals), C (cetaceans), P (pinnipeds). Table S4: Organization of *Spirometra mansoni* mitochondrial genomes. Table S5: Nucleotide composition of the protein-coding genes, tRNAs, rRNAs, and noncoding regions in the *Spirometra mansoni* mitochondrial genomes. Figure S1: Saturation analyses of transitions and transversions of mitochondrial genes used in cestode phylogeny. The *x*-axis represents the pairwise distance estimated by the GTR model, and the *y*-axis shows the absolute number of transitions (×) and transversions (△). The trend line is a best-fit 2nd-degree polynomial. Figure S2: Sliding window estimates of nucleotide diversity (*π*) along the mitochondrial genome, excluding tRNAs and noncoding regions. Figure S3: Predicted secondary structures of 22 tRNAs in the *Spirometra mansoni* mitochondrial genome from Anhui, China. Red boxes indicate differences from the Guangxi isolate. Figure S4: Predicted secondary structures of 22 tRNAs in the *Spirometra mansoni* mitochondrial genome from Guangxi, China. Red boxes indicate differences from the Anhui isolate. Figure S5: Ancestral reconstruction of life cycle host number in cestodes. Node colours indicate the probability of state occurrence, with numerical values shown only for uncertain nodes (unlabelled nodes have a probability of 1.0). Figure S6: Ancestral reconstruction of second intermediate host types in cestodes. Node colours indicate the probability of state occurrence, with numerical values shown only for uncertain nodes (unlabelled nodes have a probability of 1.0). The second intermediate host type is labelled only for taxa in which it has been clearly identified.

## References

[1] Scholz T., Kuchta R., Brabec J. (2019). Broad tapeworms (Diphyllobothriidae), parasites of wildlife and humans: Recent progress and future challenges. Int. J. Parasitol. Parasites Wildl..

[2] Fraija-Fernández N., Waeschenbach A., Briscoe A.G., Hocking S., Kuchta R., Nyman T., Littlewood D.T.J. (2021). Evolutionary transitions in broad tapeworms (Cestoda: Diphyllobothriidea) revealed by mitogenome and nuclear ribosomal operon phylogenetics. Mol. Phylogenet. Evol..

[3] Tsang H.F., Leung S.W.M., Hung T.N., Law I., Lam K.W., Chan L., Wong S.C. (2024). Molecular Identification of *Dibothriocephalus nihonkaiense* Infection Using Nanopore Sequencing: A Case Report and Literature Review. Diagnostics.

[4] Zhou K., Cao C.Y., Ru S.S., Wang R.J., Hao J., Zhang X. (2025). Transcriptomic analysis reveals that pyruvate kinase potentially plays a key role in the differentiation of *Spirometra mansoni* proglottids by regulating the glycolysis pathway. PLoS Negl. Trop. Dis..

[5] Kikuchi T., Maruyama H. (2020). Human proliferative sparganosis update. Parasitol. Int..

[6] Semenas L., Arbetman M., Viozzi G., Gentiluomo J., Bontti S. (2024). Human diphyllobothriasis in Argentina: Assessing the epidemiological significance from historical records and reports of new cases. Parasitol. Res..

[7] Waeschenbach A., Brabec J., Scholz T., Littlewood D.T.J., Kuchta R. (2017). The catholic taste of broad tapeworms—Multiple routes to human infection. Int. J. Parasitol..

[8] Kuchta R., Scholz T., Brabec J., Bray R.A. (2008). Suppression of the tapeworm order Pseudophyllidea (Platyhelminthes: Eucestoda) and the proposal of two new orders, Bothriocephalidea and Diphyllobothriidea. Int. J. Parasitol..

[9] Waeschenbach A., Webster B.L., Bray R.A., Littlewood D.T. (2007). Added resolution among ordinal level relationships of tapeworms (Platyhelminthes: Cestoda) with complete small and large subunit nuclear ribosomal RNA genes. Mol. Phylogenet. Evol..

[10] Brabec J., Kuchta R., Scholz T. (2006). Paraphyly of the Pseudophyllidea (Platyhelminthes: Cestoda): Circumscription of monophyletic clades based on phylogenetic analysis of ribosomal RNA. Int. J. Parasitol..

[11] Li W.X., Zhang D., Fu P.P., Song R., Zou H., Li M., Wu S.G., Wang G.T. (2019). Characterization and phylogenomics of the complete mitochondrial genome of the polyzoic cestode *Gangesia oligonchis* (Platyhelminthes: Onchoproteocephalidea). J. Helminthol..

[12] Li W.X., Fu P.P., Zhang D., Boyce K., Xi B.W., Zou H., Li M., Wu S.G., Wang G.T. (2018). Comparative mitogenomics supports synonymy of the genera *Ligula* and *Digramma* (Cestoda: Diphyllobothriidae). Parasit. Vectors.

[13] Li W.X., Zhang D., Boyce K., Xi B.W., Zou H., Wu S.G., Li M., Wang G.T. (2017). The complete mitochondrial DNA of three monozoic tapeworms in the Caryophyllidea: A mitogenomic perspective on the phylogeny of eucestodes. Parasit. Vectors.

[14] Cao Z.Y., Xi B.W., Li S.W., Chen K., Xie J. (2022). Characterization of the complete mitochondrial genome of *Nippotaenia mogurndae* Yamaguti and Miyata, 1940 (Cestoda: Nippotaeniidae). J. Helminthol..

[15] Kuchta R., Phillips A.J., Scholz T. (2024). Diversity and biology of *Spirometra* tapeworms (Cestoda: Diphyllobothriidea), zoonotic parasites of wildlife: A review. Int. J. Parasitol. Parasites Wildl..

[16] Eom K.S., Park H., Lee D., Choe S., Kang Y., Bia M.M., Ndosi B.A., Nath T.C., Eamudomkarn C., Keyyu J. (2019). Identity of *Spirometra theileri* from a Leopard (*Panthera pardus*) and Spotted Hyena (*Crocuta crocuta*) in Tanzania. Korean J. Parasitol..

[17] Zhang X., Duan J.Y., Wang Z.Q., Jiang P., Liu R.D., Cui J. (2017). Using the small subunit of nuclear ribosomal DNA to reveal the phylogenetic position of the plerocercoid larvae of *Spirometra* tapeworms. Exp. Parasitol..

[18] Zhang X., Duan J.Y., Shi Y.L., Jiang P., Zeng J., Wang Z.Q., Cui J. (2017). Comparative mitochondrial genomics among *Spirometra* (Cestoda: Diphyllobothriidae) and the molecular phylogeny of related tapeworms. Mol. Phylogenet. Evol..

[19] Yamasaki H., Sanpool O., Rodpai R., Sadaow L., Laummaunwai P., Un M., Thanchomnang T., Laymanivong S., Aung W.P.P., Intapan P.M. (2021). *Spirometra* species from Asia: Genetic diversity and taxonomic challenges. Parasitol. Int..

[20] Yamasaki H., Sugiyama H., Morishima Y., Kobayashi H. (2024). Description of *Spirometra asiana* sp. nov. (Cestoda: Diphyllobothriidae) found in wild boars and hound dogs in Japan. Parasitol. Int..

[21] Kuchta R., Kołodziej-Sobocińska M., Brabec J., Młocicki D., Sałamatin R., Scholz T. (2021). Sparganosis (*Spirometra*) in Europe in the Molecular Era. Clin. Infect. Dis..

[22] Xu F.F., Chen W.Q., Liu W., Liu S.S., Wang Y.X., Chen J., Cui J., Zhang X. (2022). Genetic structure of *Spirometra mansoni* (Cestoda: Diphyllobothriidae) populations in China revealed by a Target SSR-seq method. Parasit. Vectors.

[23] Zhang X., Wang H., Cui J., Jiang P., Lin M.L., Zhang Y.L., Liu R.D., Wang Z.Q. (2016). The phylogenetic diversity of *Spirometra erinaceieuropaei* isolates from southwest China revealed by multi genes. Acta Trop..

[24] Xie Y., Li Y., Gu X., Zhang S., Liu Y., Wang L., Zheng Y., Zhou X., Zuo Z., Yang G. (2019). Characterization of the complete mitochondrial genome of *Spirometra decipiens* (Cestoda: Diphyllobothriidae) from China. Mitochondrial DNA B Resour..

[25] Jeon H.K., Park H., Lee D., Choe S., Kang Y., Bia M.M., Lee S.H., Eom K.S. (2019). Complete Sequence of the Mitochondrial Genome of *Spirometra ranarum*: Comparison with *S. erinaceieuropaei* and *S. decipiens*. Korean J. Parasitol..

[26] Zhang X., Cui J., Liu L.N., Jiang P., Wang H., Qi X., Wu X.Q., Wang Z.Q. (2015). Genetic structure analysis of *Spirometra erinaceieuropaei* isolates from central and southern China. PLoS ONE.

[27] Chen S. (2023). Ultrafast one-pass FASTQ data preprocessing, quality control, and deduplication using fastp. Imeta.

[28] Bankevich A., Nurk S., Antipov D., Gurevich A.A., Dvorkin M., Kulikov A.S., Lesin V.M., Nikolenko S.I., Pham S., Prjibelski A.D. (2012). SPAdes: A new genome assembly algorithm and its applications to single-cell sequencing. J. Comput. Biol..

[29] Gruber A.R., Bernhart S.H., Lorenz R. (2015). The ViennaRNA web services. Methods Mol. Biol..

[30] Reuter J.S., Mathews D.H. (2010). RNAstructure: Software for RNA secondary structure prediction and analysis. BMC Bioinform..

[31] Zhang D., Gao F., Jakovlić I., Zou H., Zhang J., Li W.X., Wang G.T. (2020). PhyloSuite: An integrated and scalable desktop platform for streamlined molecular sequence data management and evolutionary phylogenetics studies. Mol. Ecol. Resour..

[32] Bernt M., Merkle D., Ramsch K., Fritzsch G., Perseke M., Bernhard D., Schlegel M., Stadler P.F., Middendorf M. (2007). CREx: Inferring genomic rearrangements based on common intervals. Bioinformatics.

[33] Katoh K., Standley D.M. (2013). MAFFT multiple sequence alignment software version 7: Improvements in performance and usability. Mol. Biol. Evol..

[34] Capella-Gutiérrez S., Silla-Martínez J.M., Gabaldón T. (2009). trimAl: A tool for automated alignment trimming in large-scale phylogenetic analyses. Bioinformatics.

[35] Talavera G., Castresana J. (2007). Improvement of phylogenies after removing divergent and ambiguously aligned blocks from protein sequence alignments. Syst. Biol..

[36] Xia X. (2018). DAMBE7: New and Improved Tools for Data Analysis in Molecular Biology and Evolution. Mol. Biol. Evol..

[37] Kalyaanamoorthy S., Minh B.Q., Wong T.K.F., von Haeseler A., Jermiin L.S. (2017). ModelFinder: Fast model selection for accurate phylogenetic estimates. Nat. Methods.

[38] Nguyen L.T., Schmidt H.A., von Haeseler A., Minh B.Q. (2015). IQ-TREE: A fast and effective stochastic algorithm for estimating maximum-likelihood phylogenies. Mol. Biol. Evol..

[39] Ronquist F., Teslenko M., van der Mark P., Ayres D.L., Darling A., Höhna S., Larget B., Liu L., Suchard M.A., Huelsenbeck J.P. (2012). MrBayes 3.2: Efficient Bayesian phylogenetic inference and model choice across a large model space. Syst. Biol..

[40] Letunic I., Bork P. (2021). Interactive Tree Of Life (iTOL) v5: An online tool for phylogenetic tree display and annotation. Nucleic Acids Res..

[41] Librado P., Rozas J. (2009). DnaSP v5: A software for comprehensive analysis of DNA polymorphism data. Bioinformatics.

[42] Puillandre N., Brouillet S., Achaz G. (2021). ASAP: Assemble species by automatic partitioning. Mol. Ecol. Resour..

[43] Zhao C.H., Yang R.J., Ru S.S., Chen H.X., Li D.X., Li L. (2024). Integrative taxonomy of the genus *Pseudoacanthocephalus* (Acanthocephala: Echinorhynchida) in China, with the description of two new species and the characterization of the mitochondrial genomes of *Pseudoacanthocephalus sichuanensis* sp. n. and *Pseudoacanthocephalus nguyenthileae*. Parasit. Vectors.

[44] Katoh T.K., Chen J.M., Yang J.H., Zhang G., Wang L., Suwito A., Ak Meleng P., Toda M.J., Zhang Y.P., Gao J.J. (2024). Molecular phylogeny and species diversity of the genus *Dichaetophora* Duda and related taxa (Diptera: Drosophilidae). Mol. Phylogenet. Evol..

[45] Gu X.H., Chen H.X., Hu J.J., Li L. (2024). Morphology and ASAP analysis of the important zoonotic nematode parasite *Baylisascaris procyonis* (Stefahski and Zarnowski, 1951), with molecular phylogenetic relationships of *Baylisascaris species* (Nematoda: Ascaridida). Parasitology.

[46] Chevenet F., Castel G., Jousselin E., Gascuel O. (2019). PastView: A user-friendly interface to explore ancestral scenarios. BMC Evol. Biol..

[47] Bouckaert R., Vaughan T.G., Barido-Sottani J., Duchêne S., Fourment M., Gavryushkina A., Heled J., Jones G., Kühnert D., De Maio N. (2019). BEAST 2.5: An advanced software platform for Bayesian evolutionary analysis. PLoS Comput. Biol..

[48] Knapp J., Nakao M., Yanagida T., Okamoto M., Saarma U., Lavikainen A., Ito A. (2011). Phylogenetic relationships within *Echinococcus* and *Taenia* tapeworms (Cestoda: Taeniidae): An inference from nuclear protein-coding genes. Mol. Phylogenet. Evol..

[49] Attwood S.W., Fatih F.A., Upatham E.S. (2008). DNA-sequence variation among *Schistosoma mekongi* populations and related taxa; phylogeography and the current distribution of Asian schistosomiasis. PLoS Negl. Trop. Dis..

[50] Rambaut A., Drummond A.J., Xie D., Baele G., Suchard M.A. (2018). Posterior Summarization in Bayesian Phylogenetics Using Tracer 1.7. Syst. Biol..

[51] Ricklefs R.E. (2007). Estimating diversification rates from phylogenetic information. Trends Ecol. Evol..

[52] Zhang X., Wang H., Cui J., Jiang P., Fu G.M., Zhong K., Zhang Z.F., Wang Z.Q. (2015). Characterisation of the relationship between *Spirometra erinaceieuropaei* and *Diphyllobothrium* species using complete *cytb* and *cox1* genes. Infect. Genet. Evol..

[53] Xing B., Lin L., Wu Q. (2025). Application of mitochondrial genomes to species identification and evolution. Electron. J. Biotechnol..

[54] Ferreira T., Rodriguez S. (2024). Mitochondrial DNA: Inherent Complexities Relevant to Genetic Analyses. Genes.

[55] Ontano A.Z., Gainett G., Aharon S., Ballesteros J.A., Benavides L.R., Corbett K.F., Gavish-Regev E., Harvey M.S., Monsma S., Santibáñez-López C.E. (2021). Taxonomic Sampling and Rare Genomic Changes Overcome Long-Branch Attraction in the Phylogenetic Placement of Pseudoscorpions. Mol. Biol. Evol..

[56] Waeschenbach A., Webster B.L., Littlewood D.T. (2012). Adding resolution to ordinal level relationships of tapeworms (Platyhelminthes: Cestoda) with large fragments of mtDNA. Mol. Phylogenet. Evol..

[57] Hernández-Orts J.S., Kuzmina T.A., Gomez-Puerta L.A., Kuchta R. (2021). *Diphyllobothrium sprakeri* n. sp. (Cestoda: Diphyllobothriidae): A hidden broad tapeworm from sea lions off North and South America. Parasit. Vectors.

[58] Yamasaki H., Ohmae H., Kuramochi T. (2012). Complete mitochondrial genomes of *Diplogonoporus balaenopterae* and *Diplogonoporus grandis* (Cestoda: Diphyllobothriidae) and clarification of their taxonomic relationships. Parasitol. Int..

[59] Guo A. (2016). The complete mitochondrial genome of the tapeworm *Cladotaenia vulturi* (Cestoda: Paruterinidae): Gene arrangement and phylogenetic relationships with other cestodes. Parasit. Vectors.

[60] Vettorazzi R., Norbis W., Martorelli S.R., Garcia G., Rios N. (2023). First report of *Spirometra* (Eucestoda; Diphyllobothriidae) naturally occurring in a fish host. Folia Parasitol..

[61] Rubinoff D., Cameron S., Will K. (2006). A genomic perspective on the shortcomings of mitochondrial DNA for “barcoding” identification. J. Hered..

[62] Dai G., Su Y., Li L., Chen W., Li L., Chen F., Ni G., Wang G., Xue W., Bai P. (2025). Mitochondrial genome characterization and phylogenetic position of a putative taeniid tapeworm (*Taenia* sp. QH-2023) isolated from Pseudois nayaur. Acta Trop..

[63] Levinger L., Serjanov D. (2012). Pathogenesis-related mutations in the T-loops of human mitochondrial tRNAs affect 3′ end processing and tRNA structure. RNA Biol..

[64] Scholz T., Waeschenbach A., Oros M., Brabec J., Littlewood D.T.J. (2021). Phylogenetic reconstruction of early diverging tapeworms (Cestoda: Caryophyllidea) reveals ancient radiations in vertebrate hosts and biogeographic regions. Int. J. Parasitol..

[65] Mendlovic F., Garza-Rodríguez A., Carrillo-Farga J., González-Domínguez F., Maravilla P., Flisser A. (2014). From stillness to motion: 80 years after the first description of *Taenia solium* oncosphere hatching. Parasit. Vectors.

[66] Michelet L., Dauga C. (2012). Molecular evidence of host influences on the evolution and spread of human tapeworms. Biol. Rev. Camb. Philos. Soc..

[67] Cislo P.R., Caira J.N. (1993). The parasite assemblage in the spiral intestine of the shark *Mustelus canis*. J. Parasitol..

[68] Nagy J. (2020). Biologia Futura: Rapid diversification and behavioural adaptation of birds in response to Oligocene-Miocene climatic conditions. Biol. Futur..

[69] Janis C.M., Damuth J., Theodor J.M. (2000). Miocene ungulates and terrestrial primary productivity: Where have all the browsers gone?. Proc. Natl. Acad. Sci. USA.

[70] Edwards E.J., Osborne C.P., Strömberg C.A., Smith S.A., Bond W.J., Christin P.A., Cousins A.B., Duvall M.R., Fox D.L., Freckleton R.P. (2010). The origins of C4 grasslands: Integrating evolutionary and ecosystem science. Science.

[71] Hoberg E.P., Alkire N.L., de Queiroz A., Jones A. (2001). Out of Africa: Origins of the *Taenia* tapeworms in humans. Proc. Biol. Sci..

[72] Hoberg E.P. (2006). Phylogeny of *Taenia*: Species definitions and origins of human parasites. Parasitol. Int..

[73] Domínguez-Rodrigo M., Pickering T.R., Semaw S., Rogers M.J. (2005). Cutmarked bones from Pliocene archaeological sites at Gona, Afar, Ethiopia: Implications for the function of the world’s oldest stone tools. J. Hum. Evol..

