# Supplementary material for: Phylogenetic Inference and Ancestral Character Reconstruction of Diphyllobothriid Tapeworms (Cestoda: Diphyllobothriidae)
Source: Animals (Basel). 2026 Jul 6;16(13):2084. doi: 10.3390/ani16132084 (PMC13360106; doi:10.3390/ani16132084)
Supplement: Supplementary file 1 [file animals-16-02084-s001.zip › animals-4407137-supplementary.pdf]

Supplementary materials

Phylogenetic Inference and Ancestral Character Reconstruction of Diphyllobothriid Tapeworms (Cestoda: Diphyllobothriidae)

Table S1 Mitochondrial genome sequences of the cestodes used in this study. Asterisks (*) indicate newly obtained sequences.

Table S2 Best models selected under the Bayesian information criterion (BIC) for each subset partition in this study.

Table S3 Classification of cestodes based on four traits. 1 indicates that the trait is present, and 0 indicates that the trait is absent. Habitat: T (terrestrial), F (freshwater), M (marine). Number of hosts: O (one), Tw (two), Th (three). Second intermediate host: A (absent), U (unknown), F (fish), T (tetrapods). Definitive host: F (fish), A (amphibians), R (reptiles), B (birds), T (terrestrial mammals), C (cetaceans), P (pinnipeds).

Table S4 Organization of *Spirometra mansoni* mitochondrial genomes.

Table S5 Nucleotide composition of the protein-coding genes, tRNAs, rRNAs, and noncoding regions in the *Spirometra mansoni* mitochondrial genomes.

Figure S1 Saturation analyses of transitions and transversions of mitochondrial genes used in cestode phylogeny. The x-axis represents the pairwise distance estimated by the GTR model, and the y-axis shows the absolute number of transitions (×) and transversions (△). The trend line is a best-fit 2nd-degree polynomial.

Figure S2 Sliding window estimates of nucleotide diversity (π) along the mitochondrial genome, excluding tRNAs and noncoding regions.

Figure S3 Predicted secondary structures of 22 tRNAs in the *Spirometra mansoni* mitochondrial genome from Anhui, China. Red boxes indicate differences from the Guangxi isolate.

Figure S4 Predicted secondary structures of 22 tRNAs in the *Spirometra mansoni* mitochondrial genome from Guangxi, China. Red boxes indicate differences from the Anhui isolate.

Figure S5 Ancestral reconstruction of life cycle host number in cestodes. Node colours indicate the probability of state occurrence, with numerical values shown only for uncertain nodes (unlabelled nodes have a probability of 1.0).

Figure S6 Ancestral reconstruction of second intermediate host types in cestodes. Node colours indicate the probability of state occurrence, with numerical values shown only for uncertain nodes (unlabelled nodes have a probability of 1.0). The second intermediate host type is labelled only for taxa in which it has been clearly identified.

Table S1 Mitochondrial genome sequences of the cestodes used in this study. Asterisks (*) indicate newly obtained sequences.

Order	Family	Species	Host	Location	Accession number
Strigeidida	Schistosomatidae	*Schistosoma haematobium*	*Mesocricetus auratus*	Mali	NC_008074
		Schistosoma japonicum	-	China (Anhui)	NC_002544
		Schistosoma mansoni	-	USA	NC_002545
		Schistosoma mekongi	-	Laos	NC_002529
Bothriocephalidea	Bothriocephalidae	*Schyzocotyle acheilognathi*	*Labeobarbus kimberleyensis*	South Africa	KX060593
		Schyzocotyle acheilognathi	*Cyprinus carpio*	Japan	KX060590
		Schyzocotyle acheilognathi	*Cyprinella lutrensis*	USA	KX060595
		Schyzocotyle acheilognathi	*Barbus* sp.	Ethiopia	KX060588
		Schyzocotyle acheilognathi	*Cyprinus carpio*	Czech Republic	KX060592
		Schyzocotyle acheilognathi	*Atherina boyeri*	Turkey	KX060594
		Schyzocotyle acheilognathi	*Gila conspersa*	Mexico	KX060591
		Schyzocotyle acheilognathi	*Zacco platypus*	China (Hubei)	NC_030316
		Schyzocotyle acheilognathi	*Ctenopharyngodon idella*	China (Hubei)	KX589243
		Schyzocotyle nayarensis	*Raiamas bola*	India	NC_030317
		Senga ophiocephalina	*Labeobarbus kimberleyensis*	China (Yunnan)	NC_034715
Caryophyllidea	Capingentidae	*Breviscolex orientalis*	*Hemibarbus maculates*	China (Hubei)	NC_035634
	Caryophyllaeidae	*Caryophyllaeus brachycollis*	*Cyprinus carpio*	China	NC_035430
		Parabreviscolex niepini	*Schizopygopsis*	China (Tibet)	NC_040117
	Lytocestidae	*Atractolytocestus huronensis*	*Cyprinus carpio*	China (Hubei)	NC_035635
		Khawia sinensis	*Carassius auratus*	China (Hubei)	NC_034800
Cyclophyllidea	Anoplocephalidae	*Anoplocephala magna*	Donkey	China	NC_031801
		Anoplocephala perfoliata	Donkey	China	NC_028425
		Moniezia benedeni	*buffalo calves*	China (Guangxi)	NC_036218

	Moniezia expansa	*buffalo calves*	China (Guangxi)	NC_036219
	Mosgovoyia sp. SQ20	*Ochotona curzoniae*	China	NC_061207
	Paranoplocephala sp. RKZ13	*Neodon irene*	China	NC_061205
Dipylidiidae	*Dipylidium caninum*	-	Unknown	NC_021145
Hymenolepididae	*Cloacotaenia megalops*	Wild duck	China (Xingjiang)	NC_032295
	Drepanidotaenia lanceolata	Rhine geese	China	NC_028164
	Hymenolepis diminuta	Rat	-	NC_002767
	Hymenolepis microstoma	*Mus musculus*	-	LC102493
	Pseudanoplocephala crawfordi	Pig	China (Shaanxi)	NC_028334
	Rodentolepis nana	Mouse	China (Gansu)	NC_029245
Mesocestoididae	*Mesocestoides corti*	-	-	AP017667
	Mesocestoides vogae	*Mus musculus*	-	LC102498
Nematotaeniidae	*Cylindrotaenia japonica*	*Zhangixalus arboreus*	Japan	LC710154
Paruterinidae	*Cladotaenia vulturi*	*Aquila nipalensis*	China	NC_032067
	Paruterina candelabraria	*Athene noctua*	China	NC_039533
Taeniidae	*Echinococcus canadensis*	Camel	Kazakhstan	NC_011121
	Echinococcus equinus	*Equus caballus*	United Kingdom	NC_020374
	Echinococcus felidis	-	Uganda	NC_021144
	Echinococcus granulosus	Sheep	Australia	NC_044548
	Echinococcus multilocularis	*Clethrionomys rufocanus*	Japan	NC_000928
	Echinococcus oligarthrus	Mouse	Panama	NC_009461
	Echinococcus ortleppi	Cattle	-	NC_011122
	Echinococcus shiquicus	*Ochotona curzoniae*	China	NC_009460
	Echinococcus vogeli	-	-	NC_009462
	Hydatigera kamiyai	-	Finland	NC_037071
	Hydatigera krepkogorski	-	China	NC_021142

		Hydatigera parva	-	Spain	NC_021141
		Hydatigera taeniaeformis	-	-	NC_056571
		Taenia arctos	*Ursus arctos*	Finland	NC_024590
		Taenia asiatica	-	Korea	NC_004826
		Taenia caixuepengi	*Ochotona curzoniae*	China	MT882036
		Taenia crassiceps	-	American	NC_002547
		Taenia crocutae	*Crocuta crocuta*	Ethiopia	NC_024591
		Taenia hydatigena	Sheep	China	NC_012896
		Taenia laticollis	-	Finland	NC_021140
		Taenia madoquae	-	Kenya	NC_021139
		Taenia martis	-	Croatia	NC_020153
		Taenia multiceps	Dog	China	NC_012894
		Taenia ovis	-	New Zealand	NC_021138
		Taenia pisiformis	Dog	China	NC_013844
		Taenia regis	*Panthera leo*	Kenya	NC_024589
		Taenia saginata	*Homo sapiens*	Belgian	NC_009938
		Taenia serialis	-	Australia	NC_021457
		Taenia solium	-	China	NC_004022
		Taenia tianguangfui	*Neodon fuscus*	China	MT882037
		Taenia twitchelli	-	Russia	NC_021093
		Versteria mustelae	-	Finland	NC_021143
Diphyllobothriidea	Cephalochlamydidae	*Cephalochlamys namaquensis*	*Xenopus muelleri*	South Africa	MW602522
		Cephalochlamys namaquensis	*Xenopus laevis*	USA	MW602524
	Diphyllobothriidae	*Adenocephalus pacificus*	*Homo sapiens*	Peru	MW602527
		Dibothriocephalus dendriticus	*Homo sapiens*	Czech Republic	MW602518
		Dibothriocephalus latus	-	Switzerland	AP017663

	Dibothriocephalus latus	*Homo sapiens*	Russia	NC_008945
	Dibothriocephalus latus	*Homo sapiens*	Russia	AB269325
	Dibothriocephalus nihonkaiensis	*Homo sapiens*	Korea	EF420138
	Dibothriocephalus nihonkaiensis	*Homo sapiens*	Japan	NC_009463
	Digramma interrupta	*Carassius auratus*	China (Hubei)	NC_039446
	Diphyllobothrium cordatum	*Erignathus barbatus*	USA	MW602523
	Diphyllobothrium schistochilos	*Pusa hispida*	Norway	MW602528
	Diphyllobothrium stemmacephalum	*Homo sapiens*	Japan	NC_035881
	Diplogonoporus balaenopterae	*Balaenoptera acutorostrata*	Japan	NC_017613
	Diplogonoporus grandis	*Homo sapiens*	Japan	NC_017615
	Ligula intestinalis	*Pusa hispida saimensis*	Finland	MW602520
	Ligula intestinalis	*Gymnocypris selincuoensis*	China (Tibet)	NC_039445
	Ligula intestinalis	*Pusa hispida ladogensis*	Russia	MW602519
	Ligula intestinalis	*Pseudorasbora parva*	China	PP109086
	Ligula intestinalis	*Orestias agassizii*	Peru	OR756289
	Schistocephalus pungitii	*Pungitius pungitius*	Germany	MW602516
	Schistocephalus solidus	*Gasterosteus aculeatus*	Norway	MW602517
	Schistocephalus solidus	*Pusa hispida botnica*	Finland	MW602521
	Schistocephalus solidus	-	-	AP017669
	Sparganum proliferum	-	Venezuela	NC_071928
	Spirometra decipiens	*Rhabdophis tigrinus tigrinus*	Korea	NC_026852
	Spirometra decipiens	Dog	China (Sichuan)	MN121695
	Spirometra erinaceieuropaei	-	-	KU852381
	Spirometra erinaceieuropaei	-	-	AP017668
	Spirometra erinaceieuropaei	*Rana temporaria*	China (Henan)	KY114887
	Spirometra erinaceieuropaei	*Elaphe taeniurus*	China (Hunan)	OM935775

		Spirometra erinaceieuropaei	*Ptyas dhumnades*	China (Hunan)	OM935777
		Spirometra erinaceieuropaei	*Elaphe carinata*	China (Hunan)	OM935776
		Spirometra erinaceieuropaei	-	Japan	OX421841
		Spirometra erinaceieuropaei	*Homo sapiens*	Korea	KJ599680
		Spirometra erinaceieuropaei	*Rana nigromaculata*	China (Jiangsu)	KY114886
		Spirometra erinaceieuropaei	*Elaphe quadrivirgata*	Japan	NC_011037
		Spirometra erinaceieuropaei	*Prionailurus bengalensis*	China (Hunan)	OM935780
		Spirometra erinaceieuropaei	*Panthera tigris* ssp.*tigris*	China (Hunan)	OM935779
		Spirometra erinaceieuropaei	*Felis catus*	China (Hunan)	OM935781
		Spirometra erinaceieuropaei	*Rana rugulosa*	China (Hainan)	KY114888
		Spirometra erinaceieuropaei	*Hylarana guentheri*	China (Sichuan)	KY114889
		Spirometra erinaceieuropaei	*Panthera tigris* ssp.*altaica*	China (Hunan)	OM935778
		Spirometra erinaceieuropaei	Dog	China (Guangdong)	JQ267473
		Spirometra mansoni	*Rana nigromaculata*	China (Anhui)	PX897701*
		Spirometra mansoni	*Rana limnocharis*	China (Guangxi)	PX897702*
		Spirometra ranarum	*Hoplobatrachus rugulosus*	Myanmar	NC_061250
		Spirometra theileri	*Panthera pardus*	Tanzania	NC_056327
	Solenophoridae	*Bothridium pithonis*	*Xenopeltis unicolor*	Viet Nam	MW602526
		Duthiersia expansa	*Varanus salvator*	Viet Nam	MW602525
Haplobothriidea	Haplobothriidae	*Haplobothrium globuliforme*	*Amia calva*	Canada	MW602515
Nippotaeniidea	Nippotaeniidae	*Nippotaenia mogurndae*	*Perccottus glenii*	China	NC_066810
Proteocephalidea	Proteocephalidae	*Gangesia oligonchis*	*Tachysurus fulvidraco*	China (Hubei)	NC_046386
		Testudotaenia sp. WL-2016	-	-	KU761587
Rhinebothriidea	Anthocephaliidae	*Alveobothrium grabatum*	*Taeniurops grabatus*	Senegal	MZ594603
		Anthocephalum cf. caira DJM-2021	*Styracura schmardae*	Panama	MZ594644
		Divaricobothrium sp. LRP 10377	*Urogymnus granulatus*	Solomon Islands	MZ594599

	Echeneibothriidae	*Echeneibothrium multiloculatum*	*Dipturus chilensis*	Chile	MZ594651
		Echeneibothrium williamsi	*Dipturus chilensis*	Chile	MZ594641
	Rhinebothriidae	*Rhabdotobothrium anterophallum*	*Mobula hypostoma*	USA	MZ594582
		Rhinebothrium flexile	*Bathytoshia centroura*	USA	MZ594571
		Rhinebothrium fulbrighti	*Potamotrygon orbignyi*	Brazil	MZ594638
		Rhinebothrium megacanthophallus	*Urogymnus polylepis*	Malaysia	MZ594574
		Rhinebothrium reydai	*Styracura schmardae*	-	NC_044703
		Rhinebothrium scobinae	*Psammobatis scobina*	Chile	MZ594607
		Rhinebothrium taeniuri	*Taeniura lymma*	Egypt	MZ594576
		Rhinebothroides sp. MZUSP 8020	*Potamotrygon wallacei*	Brazil	MZ594615
		Rhodobothrium cf. paucitesticulare DJM-2021	*Rhinoptera* sp.	Brazil	MZ594637
		Scalithrium magniphalum	*Styracura schmardae*	Panama	MZ594631
		Spongiobothrium variabile	*Bathytoshia centroura*	USA	MZ594570
Spathebothriidea	Acrobothriidae	*Didymobothrium rudolphii*	*Pegusa lascaris*	Portugal	MW602514
Tetraphyllidea	Onchobothriidae	*Acanthobothrium* sp. MZUSP 7998	*Hypanus longus*	Panama	MZ594642
		Potamotrygonocestus sp. MZUSP 7996	*Potamotrygon orbignyi*	Brazil	MZ594639
	Serendipidae	*Duplicibothrium* sp. MZUSP 8039	*Rhinoptera* sp.	Brazil	MZ594652
		Serendip sp. MZUSP 7987	*Aetomylaeus asperrimus*	Panama	MZ594632
Trypanorhyncha	Eutetrarhynchidae	*Dollfusiella* sp. MZUSP 7992	*Urotrygon aspidura*	Panama	MZ594636

Table S2 Best models selected under the Bayesian information criterion (BIC) for each subset partition in this study.

Datasets	Software	Methods	Subset partitions	Best model
Nucleotide sequences of 12 PCGs	IQ-TREE v2.2.0	ML	P1: *atp*6, *cox*1, *cox*2, *cox*3, *cytb*, *nad*1, *nad*2, *nad*3, *nad*4L, *nad*4, *nad*5, *nad*6	TVM+F+I+I+R10
	MrBayes v3.2.7	BI	P1: *atp*6, nad5<br>P2: *cox*1<br>P3: *cox*2<br>P4: *cox*3, *nad*3, *nad*6<br>P5: *cytb*<br>P6: *nad*1, *nad*2<br>P7: *nad*4<br>P8: *nad*4L	P1-P7: GTR+F+I+G4<br>P8: GTR+F+G4
Nucleotide sequences of 12 PCGs and 2 rRNAs	BEAST v2.7.7	BI	P1: *atp*6, *cox*2, *cytb*, *nad*4L<br>P2: *cox*1<br>P3: *cox*3, *nad*3, *nad*6<br>P4: *nad*1, *nad*2<br>P5: *nad*4<br>P6: *nad*5<br>P7: *rrnL*<br>P8: *rrnS*	P1-P8: GTR+F+I+G4

Table S3 Classification of cestodes based on four traits. 1 indicates that the trait is present, and 0 indicates that the trait is absent. Habitat: T (terrestrial), F (freshwater), M (marine). Number of hosts: O (one), Tw (two), Th (three). Second intermediate host: A (absent), U (unknown), F (fish), T (tetrapods). Definitive host: F (fish), A (amphibians), R (reptiles), B (birds), T (terrestrial mammals), C (cetaceans), P (pinnipeds).

**Species**	**Accession number**	**Habitat**			**Number of hosts**			**Second intermediate host**				**Definitive host**						
		**T**	**F**	**M**	**O**	**Tw**	**Th**	**A**	**U**	**F**	**T**	**F**	**A**	**R**	**B**	**T**	**C**	**P**
Schistosoma mansoni	NC_002545	0	1	0	0	1	0	1	0	0	0	0	0	0	0	1	0	0
Schistosoma haematobium	NC_008074	0	1	0	0	1	0	1	0	0	0	0	0	0	0	1	0	0
Schistosoma mekongi	NC_002529	0	1	0	0	1	0	1	0	0	0	0	0	0	0	1	0	0
Schistosoma japonicum	NC_002544	0	1	0	0	1	0	1	0	0	0	0	0	0	0	1	0	0
Khawia sinensis	NC_034800	0	1	0	0	1	0	1	0	0	0	1	0	0	0	0	0	0
Caryophyllaeus brachycollis	NC_035430	0	1	0	0	1	0	1	0	0	0	1	0	0	0	0	0	0
Parabreviscolex niepini	NC_040117	0	1	0	0	1	0	1	0	0	0	1	0	0	0	0	0	0
Breviscolex orientalis	NC_035634	0	1	0	0	1	0	1	0	0	0	1	0	0	0	0	0	0
Atractolytocestus huronensis	NC_035635	0	1	0	0	1	0	1	0	0	0	1	0	0	0	0	0	0
Didymobothrium rudolphii	MW602514	0	0	1	0	1	0	1	0	0	0	1	0	0	0	0	0	0
Haplobothrium globuliforme	MW602515	0	1	0	0	1	0	1	0	0	0	1	0	0	0	0	0	0
Cephalochlamys namaquensis	MW602524	0	1	0	0	1	0	1	0	0	0	0	1	0	0	0	0	0
Cephalochlamys namaquensis	MW602522	0	1	0	0	1	0	1	0	0	0	0	1	0	0	0	0	0
Duthiersia expansa	MW602525	0	1	0	0	0	1	0	1	0	0	0	0	1	0	0	0	0
Bothridium pithonis	MW602526	0	1	0	0	0	1	0	1	0	0	0	0	1	0	0	0	0
Schistocephalus pungitii	MW602516	0	1	0	0	0	1	0	0	1	0	0	0	0	1	0	0	0
Schistocephalus solidus	AP017669	0	1	0	0	0	1	0	0	1	0	0	0	0	1	0	0	0
Schistocephalus solidus	MW602521	0	1	0	0	0	1	0	0	1	0	0	0	0	1	0	0	0
Schistocephalus solidus	MW602517	0	1	0	0	0	1	0	0	1	0	0	0	0	1	0	0	0
Adenocephalus pacificus	MW602527	0	0	1	0	0	1	0	0	1	0	0	0	0	0	0	0	1

Diphyllobothrium stemmacephalum	NC_035881	0	0	1	0	0	1	0	0	1	0	0	0	0	0	0	1	0
Diplogonoporus grandis	NC_017615	0	0	1	0	0	1	0	0	1	0	0	0	0	0	0	1	0
Diplogonoporus balaenopterae	NC_017613	0	0	1	0	0	1	0	0	1	0	0	0	0	0	0	1	0
Diphyllobothrium cordatum	MW602523	0	0	1	0	0	1	0	0	1	0	0	0	0	0	0	0	1
Diphyllobothrium schistochilos	MW602528	0	0	1	0	0	1	0	0	1	0	0	0	0	0	0	0	1
Ligula intestinalis	OR756289	0	1	0	0	0	1	0	0	1	0	0	0	0	1	0	0	0
Ligula intestinalis	PP109086	0	1	0	0	0	1	0	0	1	0	0	0	0	1	0	0	0
Ligula intestinalis	NC_039445	0	1	0	0	0	1	0	0	1	0	0	0	0	1	0	0	0
Digramma interrupta	NC_039446	0	1	0	0	0	1	0	0	1	0	0	0	0	1	0	0	0
Ligula intestinalis	MW602520	0	1	0	0	0	1	0	0	1	0	0	0	0	1	0	0	0
Ligula intestinalis	MW602519	0	1	0	0	0	1	0	0	1	0	0	0	0	1	0	0	0
Dibothriocephalus nihonkaiensis	NC_009463	0	1	0	0	0	1	0	0	1	0	0	0	0	0	1	0	0
Dibothriocephalus nihonkaiensis	EF420138	0	1	0	0	0	1	0	0	1	0	0	0	0	0	1	0	0
Dibothriocephalus dendriticus	MW602518	0	1	0	0	0	1	0	0	1	0	0	0	0	1	0	0	0
Dibothriocephalus latus	AB269325	0	1	0	0	0	1	0	0	1	0	0	0	0	0	1	0	0
Dibothriocephalus latus	NC_008945	0	1	0	0	0	1	0	0	1	0	0	0	0	0	1	0	0
Dibothriocephalus latus	AP017663	0	1	0	0	0	1	0	0	1	0	0	0	0	0	1	0	0
Sparganum proliferum	NC_071928	0	1	0	0	0	1	0	0	0	1	0	0	0	0	1	0	0
Spirometra theileri	NC_056327	0	1	0	0	0	1	0	0	0	1	0	0	0	0	1	0	0
Spirometra erinaceieuropaei	KJ599680	0	1	0	0	0	1	0	0	0	1	0	0	0	0	1	0	0
Spirometra erinaceieuropaei	OX421841	0	1	0	0	0	1	0	0	0	1	0	0	0	0	1	0	0
Spirometra mansoni	PX897702	0	1	0	0	0	1	0	0	0	1	0	0	0	0	1	0	0
Spirometra erinaceieuropaei	AP017668	0	1	0	0	0	1	0	0	0	1	0	0	0	0	1	0	0
Spirometra ranarum	NC_061250	0	1	0	0	0	1	0	0	0	1	0	0	0	0	1	0	0
Spirometra erinaceieuropaei	OM935779	0	1	0	0	0	1	0	0	0	1	0	0	0	0	1	0	0
Spirometra erinaceieuropaei	OM935775	0	1	0	0	0	1	0	0	0	1	0	0	0	0	1	0	0
Spirometra erinaceieuropaei	JQ267473	0	1	0	0	0	1	0	0	0	1	0	0	0	0	1	0	0

Spirometra erinaceieuropaei	OM935777	0	1	0	0	0	1	0	0	0	1	0	0	0	0	1	0	0
Spirometra erinaceieuropaei	OM935776	0	1	0	0	0	1	0	0	0	1	0	0	0	0	1	0	0
Spirometra erinaceieuropaei	KY114888	0	1	0	0	0	1	0	0	0	1	0	0	0	0	1	0	0
Spirometra erinaceieuropaei	KY114889	0	1	0	0	0	1	0	0	0	1	0	0	0	0	1	0	0
Spirometra decipiens	NC_026852	0	1	0	0	0	1	0	0	0	1	0	0	0	0	1	0	0
Spirometra decipiens	MN121695	0	1	0	0	0	1	0	0	0	1	0	0	0	0	1	0	0
Spirometra erinaceieuropaei	NC_011037	0	1	0	0	0	1	0	0	0	1	0	0	0	0	1	0	0
Spirometra erinaceieuropaei	KY114886	0	1	0	0	0	1	0	0	0	1	0	0	0	0	1	0	0
Spirometra mansoni	PX897701	0	1	0	0	0	1	0	0	0	1	0	0	0	0	1	0	0
Spirometra erinaceieuropaei	KY114887	0	1	0	0	0	1	0	0	0	1	0	0	0	0	1	0	0
Spirometra erinaceieuropaei	OM935780	0	1	0	0	0	1	0	0	0	1	0	0	0	0	1	0	0
Spirometra erinaceieuropaei	OM935778	0	1	0	0	0	1	0	0	0	1	0	0	0	0	1	0	0
Spirometra erinaceieuropaei	OM935781	0	1	0	0	0	1	0	0	0	1	0	0	0	0	1	0	0
Spirometra erinaceieuropaei	KU852381	0	1	0	0	0	1	0	0	0	1	0	0	0	0	1	0	0
Dollfusiella sp. MZUSP 7992	MZ594636	0	0	1	0	1	0	1	0	0	0	1	0	0	0	0	0	0
Senga ophiocephalina	NC_034715	0	1	0	0	1	0	1	0	0	0	1	0	0	0	0	0	0
Schyzocotyle nayarensis	NC_030317	0	1	0	0	1	0	1	0	0	0	1	0	0	0	0	0	0
Schyzocotyle acheilognathi	KX060594	0	1	0	0	1	0	1	0	0	0	1	0	0	0	0	0	0
Schyzocotyle acheilognathi	KX060588	0	1	0	0	1	0	1	0	0	0	1	0	0	0	0	0	0
Schyzocotyle acheilognathi	KX589243	0	1	0	0	1	0	1	0	0	0	1	0	0	0	0	0	0
Schyzocotyle acheilognathi	KX060590	0	1	0	0	1	0	1	0	0	0	1	0	0	0	0	0	0
Schyzocotyle acheilognathi	KX060592	0	1	0	0	1	0	1	0	0	0	1	0	0	0	0	0	0
Schyzocotyle acheilognathi	KX060593	0	1	0	0	1	0	1	0	0	0	1	0	0	0	0	0	0
Schyzocotyle acheilognathi	NC_030316	0	1	0	0	1	0	1	0	0	0	1	0	0	0	0	0	0
Schyzocotyle acheilognathi	KX060595	0	1	0	0	1	0	1	0	0	0	1	0	0	0	0	0	0
Schyzocotyle acheilognathi	KX060591	0	1	0	0	1	0	1	0	0	0	1	0	0	0	0	0	0
Serendip sp. MZUSP 7987	MZ594632	0	0	1	0	1	0	1	0	0	0	1	0	0	0	0	0	0

Duplicibothrium sp. MZUSP 8039	MZ594652	0	0	1	0	1	0	1	0	0	0	1	0	0	0	0	0	0
Testudotaenia sp. WL-2016	KU761587	0	1	0	0	0	1	0	1	0	0	0	0	1	0	0	0	0
Gangesia oligonchis	NC_046386	0	1	0	0	1	0	1	0	0	0	1	0	0	0	0	0	0
Potamotrygonocestus sp. MZUSP 7996	MZ594639	0	1	0	0	1	0	1	0	0	0	1	0	0	0	0	0	0
Acanthobothrium sp. MZUSP 7998	MZ594642	0	0	1	0	1	0	1	0	0	0	1	0	0	0	0	0	0
Divaricobothrium sp. LRP 10377	MZ594599	0	0	1	0	1	0	1	0	0	0	1	0	0	0	0	0	0
Anthocephalum cf. caira DJM-2021	MZ594644	0	0	1	0	1	0	1	0	0	0	1	0	0	0	0	0	0
Alveobothrium grabatum	MZ594603	0	0	1	0	1	0	1	0	0	0	1	0	0	0	0	0	0
Scalithrium magniphalum	MZ594631	0	0	1	0	1	0	1	0	0	0	1	0	0	0	0	0	0
Rhinebothrium scobinae	MZ594607	0	0	1	0	1	0	1	0	0	0	1	0	0	0	0	0	0
Echeneibothrium williamsi	MZ594641	0	0	1	0	1	0	1	0	0	0	1	0	0	0	0	0	0
Echeneibothrium multiloculatum	MZ594651	0	0	1	0	1	0	1	0	0	0	1	0	0	0	0	0	0
Spongiobothrium variabile	MZ594570	0	0	1	0	1	0	1	0	0	0	1	0	0	0	0	0	0
Rhodobothrium cf. paucitesticulare DJM-2021	MZ594637	0	0	1	0	1	0	1	0	0	0	1	0	0	0	0	0	0
Rhabdotobothrium anterophallum	MZ594582	0	0	1	0	1	0	1	0	0	0	1	0	0	0	0	0	0
Rhinebothrium reydai	NC_044703	0	0	1	0	1	0	1	0	0	0	1	0	0	0	0	0	0
Rhinebothrium taeniuri	MZ594576	0	0	1	0	1	0	1	0	0	0	1	0	0	0	0	0	0
Rhinebothrium megacanthophallus	MZ594574	0	1	0	0	1	0	1	0	0	0	1	0	0	0	0	0	0
Rhinebothrium flexile	MZ594571	0	0	1	0	1	0	1	0	0	0	1	0	0	0	0	0	0
Rhinebothroides sp. MZUSP 8020	MZ594615	0	1	0	0	1	0	1	0	0	0	1	0	0	0	0	0	0
Rhinebothrium fulbrighti	MZ594638	0	1	0	0	1	0	1	0	0	0	1	0	0	0	0	0	0
Nippotaenia mogurndae	NC_066810	0	1	0	0	1	0	1	0	0	0	1	0	0	0	0	0	0
Mesocestoides vogae	LC102498	1	0	0	0	0	1	0	0	0	1	0	0	0	0	1	0	0
Mesocestoides corti	AP017667	1	0	0	0	0	1	0	0	0	1	0	0	0	0	1	0	0
Dipylidium caninum	NC_021145	1	0	0	0	1	0	1	0	0	0	0	0	0	0	1	0	0

Cloacotaenia megalops	NC_032295	0	1	0	0	1	0	1	0	0	0	0	0	0	1	0	0	0
Drepanidotaenia lanceolata	NC_028164	0	1	0	0	1	0	1	0	0	0	0	0	0	1	0	0	0
Rodentolepis nana	NC_029245	1	0	0	1	0	0	1	0	0	0	0	0	0	0	1	0	0
Hymenolepis microstoma	LC102493	1	0	0	0	1	0	1	0	0	0	0	0	0	0	1	0	0
Pseudanoplocephala crawfordi	NC_028334	1	0	0	0	1	0	1	0	0	0	0	0	0	0	1	0	0
Hymenolepis diminuta	NC_002767	1	0	0	0	1	0	1	0	0	0	0	0	0	0	1	0	0
Moniezia expansa	NC_036219	1	0	0	0	1	0	1	0	0	0	0	0	0	0	1	0	0
Moniezia benedeni	NC_036218	1	0	0	0	1	0	1	0	0	0	0	0	0	0	1	0	0
Paranoplocephala sp. RKZ13	NC_061205	1	0	0	0	1	0	1	0	0	0	0	0	0	0	1	0	0
Mosgovoyia sp. SQ20	NC_061207	1	0	0	0	1	0	1	0	0	0	0	0	0	0	1	0	0
Anoplocephala perfoliata	NC_028425	1	0	0	0	1	0	1	0	0	0	0	0	0	0	1	0	0
Anoplocephala magna	NC_031801	1	0	0	0	1	0	1	0	0	0	0	0	0	0	1	0	0
Cylindrotaenia japonica	LC710154	1	0	0	0	1	0	1	0	0	0	0	1	0	0	0	0	0
Paruterina candelabraria	NC_039533	1	0	0	0	1	0	1	0	0	0	0	0	0	1	0	0	0
Cladotaenia vulturi	NC_032067	1	0	0	0	1	0	1	0	0	0	0	0	0	1	0	0	0
Versteria mustelae	NC_021143	1	0	0	0	1	0	1	0	0	0	0	0	0	0	1	0	0
Echinococcus oligarthrus	NC_009461	1	0	0	0	1	0	1	0	0	0	0	0	0	0	1	0	0
Echinococcus vogeli	NC_009462	1	0	0	0	1	0	1	0	0	0	0	0	0	0	1	0	0
Echinococcus granulosus	NC_044548	1	0	0	0	1	0	1	0	0	0	0	0	0	0	1	0	0
Echinococcus felidis	NC_021144	1	0	0	0	1	0	1	0	0	0	0	0	0	0	1	0	0
Echinococcus ortleppi	NC_011122	1	0	0	0	1	0	1	0	0	0	0	0	0	0	1	0	0
Echinococcus canadensis	NC_011121	1	0	0	0	1	0	1	0	0	0	0	0	0	0	1	0	0
Echinococcus equinus	NC_020374	1	0	0	0	1	0	1	0	0	0	0	0	0	0	1	0	0
Echinococcus shiquicus	NC_009460	1	0	0	0	1	0	1	0	0	0	0	0	0	0	1	0	0
Echinococcus multilocularis	NC_000928	1	0	0	0	1	0	1	0	0	0	0	0	0	0	1	0	0
Hydatigera parva	NC_021141	1	0	0	0	1	0	1	0	0	0	0	0	0	0	1	0	0
Hydatigera krepkogorski	NC_021142	1	0	0	0	1	0	1	0	0	0	0	0	0	0	1	0	0

Hydatigera taeniaeformis	NC_056571	1	0	0	0	1	0	1	0	0	0	0	0	0	0	1	0	0
Hydatigera kamiyai	NC_037071	1	0	0	0	1	0	1	0	0	0	0	0	0	0	1	0	0
Taenia tianguangfui	MT882037	1	0	0	0	1	0	1	0	0	0	0	0	0	0	1	0	0
Taenia crassiceps	NC_002547	1	0	0	0	1	0	1	0	0	0	0	0	0	0	1	0	0
Taenia twitchelli	NC_021093	1	0	0	0	1	0	1	0	0	0	0	0	0	0	1	0	0
Taenia martis	NC_020153	1	0	0	0	1	0	1	0	0	0	0	0	0	0	1	0	0
Taenia laticollis	NC_021140	1	0	0	0	1	0	1	0	0	0	0	0	0	0	1	0	0
Taenia pisiformis	NC_013844	1	0	0	0	1	0	1	0	0	0	0	0	0	0	1	0	0
Taenia caixuepengi	MT882036	1	0	0	0	1	0	1	0	0	0	0	0	0	0	1	0	0
Taenia regis	NC_024589	1	0	0	0	1	0	1	0	0	0	0	0	0	0	1	0	0
Taenia hydatigena	NC_012896	1	0	0	0	1	0	1	0	0	0	0	0	0	0	1	0	0
Taenia solium	NC_004022	1	0	0	0	1	0	1	0	0	0	0	0	0	0	1	0	0
Taenia arctos	NC_024590	1	0	0	0	1	0	1	0	0	0	0	0	0	0	1	0	0
Taenia ovis	NC_021138	1	0	0	0	1	0	1	0	0	0	0	0	0	0	1	0	0
Taenia multiceps	NC_012894	1	0	0	0	1	0	1	0	0	0	0	0	0	0	1	0	0
Taenia serialis	NC_021457	1	0	0	0	1	0	1	0	0	0	0	0	0	0	1	0	0
Taenia madoquae	NC_021139	1	0	0	0	1	0	1	0	0	0	0	0	0	0	1	0	0
Taenia crocutae	NC_024591	1	0	0	0	1	0	1	0	0	0	0	0	0	0	1	0	0
Taenia saginata	NC_009938	1	0	0	0	1	0	1	0	0	0	0	0	0	0	1	0	0
Taenia asiatica	NC_004826	1	0	0	0	1	0	1	0	0	0	0	0	0	0	1	0	0

Table S4 Organization of *Spirometra mansoni* mitochondrial genomes.

Gene	Position		Size	Intergenic nucleotides	Codon		Anti-codon	Identify (%)
	From	To			Start	Stop		
**AH_LA6/GX_WZ2**								
*cox*1	1	1566	1566		ATG	TAG		97.96
trnT-ACA	1557	1626	70	-10			ugu	100
rrnL	1627	2599/2598	973/972					98.56
trnC-TGC	2600/2599	2664/2663	65				gca	98.46
rrnS	2665/2664	3394/3393	730					98.90
*cox*2	3395/3394	3964/3963	570		ATG	TAA		97.54
trnE-GAA	3970/3969	4034/4033	65	5			uuc	100
*nad*6	4039/4038	4506/4505	468	4	ATG	TAA		98.08
trnY-TAC	4513/4512	4580/4579	68	6			gua	100
NCR1	4581/4580	4784/4783	204					95.61
*trnL*1-CTA	4785/4784	4852/4850	68/67				uag	98.53
*trnS*2-TCA	4855/4853	4919/4917	65	2			uga	100
*trnL*2-TTA	4924/4922	4988/4986	65	4			uaa	100
trnR-CGT	5004/5002	5060/5058	57	15			acg	100
*nad*5	5064/5062	6632/6630	1569	3	ATG	TAA		97.20
NCR2	6633/6631	6769/6766	137/136					95.45
trnG-GGA	6770/6767	6836/6833	67				ucc	98.51
*cox*3	6840/6837	7482/7479	643	3	GTG	T		98.44
trnH-CAC	7483/7480	7537/7534	55				gug	100
cytb	7555/7552	8664/8661	1110	17	ATG	TAA		97.66
*nad*4L	8669/8666	8929/8926	261	4	ATG	TAG		99.23
*nad*4	8890/8887	10143/10140	1254	-40	ATG	TAG		97.45
trnQ-CAA	10144/10141	10226/10223	83				uug	98.80
trnF-TTC	10204/10201	10267/10264	64	-23			gaa	100
trnM-ATG	10264/10261	10331/10328	68	-4			cau	98.53
*atp*6	10335/10332	10850/10847	516	3	ATG	TAA		97.87
*nad*2	10858/10855	11730/11727	873	7	ATG	TAG		98.17
trnV-GTA	11741/11738	11805/11802	65	10			uac	100
trnA-GCA	11823/11820	11883/11880	61	17			ugc	100
trnD-GAC	11889/11886	11952/11949	64	5			guc	100
*nad*1	11953/11950	12843/12840	891		ATG	TAA		97.87
trnN-AAC	12849/12846	12914/12911	66	5			guu	100
trnP-CCA	12921/12918	12985/12982	65	6			ugg	100
trnI-ATC	12990/12987	13055/13052	66	4			gau	98.48
trnK-AAG	13061/13058	13123/13120	63	5			cuu	100
*nad*3	13127/13124	13472/13469	346	3	ATG	T		97.40

*trnS*1-AGC	13473/13470	13531/13528	59				gcu	98.31
trnW-TGA	13534/13531	13599/13596	66	2			uca	100
Full mitogenome			13606 bp/13603bp					97.98

Table S5 Nucleotide composition of the protein-coding genes, tRNAs, rRNAs, and noncoding regions in the *Spirometra mansoni* mitochondrial genomes.

Regions	Size(bp)	T(U)	C	A	G	AT(%)	GC(%)	AT skew	GC skew
**AH_LA6**									
PCGs	10065	48.7	10.6	18.2	22.5	66.9	33.1	-0.455	0.361
1st codon position	3355	43.4	10.2	21	25.5	64.4	35.7	-0.347	0.429
2nd codon position	3355	48.1	14.8	16.8	20.4	64.9	35.2	-0.483	0.161
3rd codon position	3355	54.7	6.8	17	21.5	71.7	28.3	-0.527	0.522
*atp*6	516	51.4	10.5	16.3	21.9	67.7	32.4	-0.519	0.353
*cox*1	1566	48	11.5	17.6	22.9	65.6	34.4	-0.463	0.332
*cox*2	570	43.5	10.4	21.9	24.2	65.4	34.6	-0.33	0.401
*cox*3	643	50.5	10.3	17.6	21.6	68.1	31.9	-0.484	0.356
cytb	1110	46.5	11.4	18.4	23.7	64.9	35.1	-0.433	0.349
*nad*1	891	47.6	9	17.3	26.2	64.9	35.2	-0.467	0.489
*nad*2	873	50.5	9.9	17.5	22.1	68	32	-0.485	0.384
*nad*3	346	54.6	7.2	15.3	22.8	69.9	30	-0.562	0.519
*nad*4	1254	50.8	11.6	17.1	20.4	67.9	32	-0.495	0.274
*nad*4L	261	51.7	9.2	19.2	19.9	70.9	29.1	-0.459	0.368
*nad*5	1569	46.9	11	20.7	21.4	67.6	32.4	-0.389	0.32
*nad*6	468	51.1	9.2	18.2	21.6	69.3	30.8	-0.475	0.403
rrnL	973	37.5	12.3	25.4	24.8	62.9	37.1	-0.193	0.335
rrnS	730	37.9	13.6	25.1	23.4	63	37	-0.204	0.267
rRNAs	1703	37.7	12.9	25.2	24.2	62.9	37.1	-0.198	0.306
tRNAs	1435	39.8	11.8	26.5	22	66.3	33.8	-0.201	0.302
NCR1	204	35.3	12.3	36.3	16.2	71.6	28.5	0.014	0.138
NCR2	137	37.2	9.5	27	26.3	64.2	35.8	-0.159	0.469
Full mitogenome	13606	46.1	11	20.3	22.6	66.4	33.6	-0.388	0.347
**GX_WZ2**									
PCGs	10065	48.4	10.9	18.3	22.5	66.7	33.4	-0.452	0.348
1st codon position	3355	43.2	10.3	21.2	25.3	64.4	35.6	-0.342	0.42
2nd codon position	3355	48	14.8	16.7	20.5	64.7	35.3	-0.484	0.162
3rd codon position	3355	53.9	7.5	16.9	21.7	70.8	29.2	-0.523	0.484
*atp*6	516	50.6	11.4	16.1	21.9	66.7	33.3	-0.517	0.314
*cox*1	1566	47.9	11.6	17.5	23.1	65.4	34.7	-0.465	0.332
*cox*2	570	41.4	11.9	21.9	24.7	63.3	36.6	-0.307	0.349
*cox*3	643	50.9	10.1	17.3	21.8	68.2	31.9	-0.493	0.366
cytb	1110	45.8	12.3	18.8	23.2	64.6	35.5	-0.417	0.308
*nad*1	891	47.5	9.1	16.7	26.7	64.2	35.8	-0.479	0.492
*nad*2	873	50.1	10.2	17.2	22.6	67.3	32.8	-0.489	0.378
*nad*3	346	54.3	7.2	16.8	21.7	71.1	28.9	-0.528	0.5

*nad*4	1254	50.4	12	17.1	20.6	67.5	32.6	-0.494	0.265
*nad*4L	261	52.1	8.8	19.5	19.5	71.6	28.3	-0.455	0.378
*nad*5	1569	46.7	11.2	21	21	67.7	32.2	-0.379	0.304
*nad*6	468	51.3	9	17.7	22	69	31	-0.486	0.421
rrnL	972	37.7	12.2	25.4	24.7	63.1	36.9	-0.194	0.337
rrnS	730	38.6	12.9	24.7	23.8	63.3	36.7	-0.221	0.299
rRNAs	1702	38.1	12.5	25.1	24.3	63.2	36.8	-0.206	0.321
tRNAs	1434	39.7	11.9	26.5	21.9	66.2	33.8	-0.2	0.298
NCR1	204	34.8	13.2	36.3	15.7	71.1	28.9	0.021	0.085
NCR2	136	36	10.3	27.2	26.5	63.2	36.8	-0.14	0.44
Full mitogenome	13603	45.9	11.2	20.3	22.6	66.2	33.8	-0.386	0.339

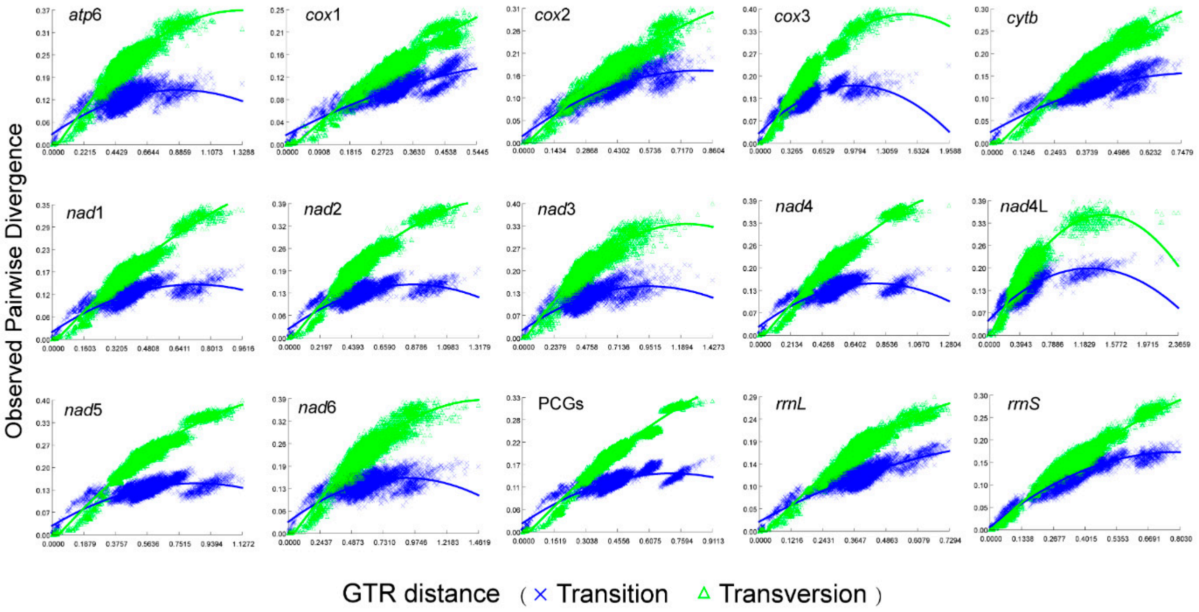

Figure S1 Saturation analyses of transitions and transversions of mitochondrial genes used in cestode phylogeny. The x-axis represents the pairwise distance estimated by the GTR model, and the y-axis shows the absolute number of transitions (×) and transversions (△). The trend line is a best-fit 2nd-degree polynomial.

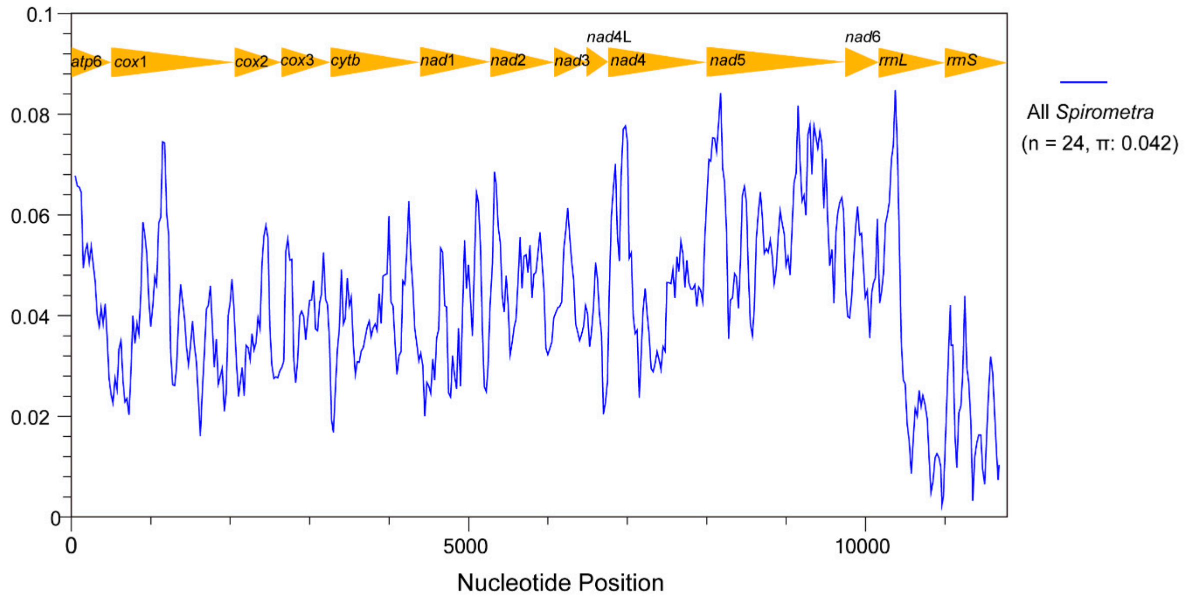

Figure S2 Sliding window estimates of nucleotide diversity (π) along the mitochondrial genome, excluding tRNAs and noncoding regions.

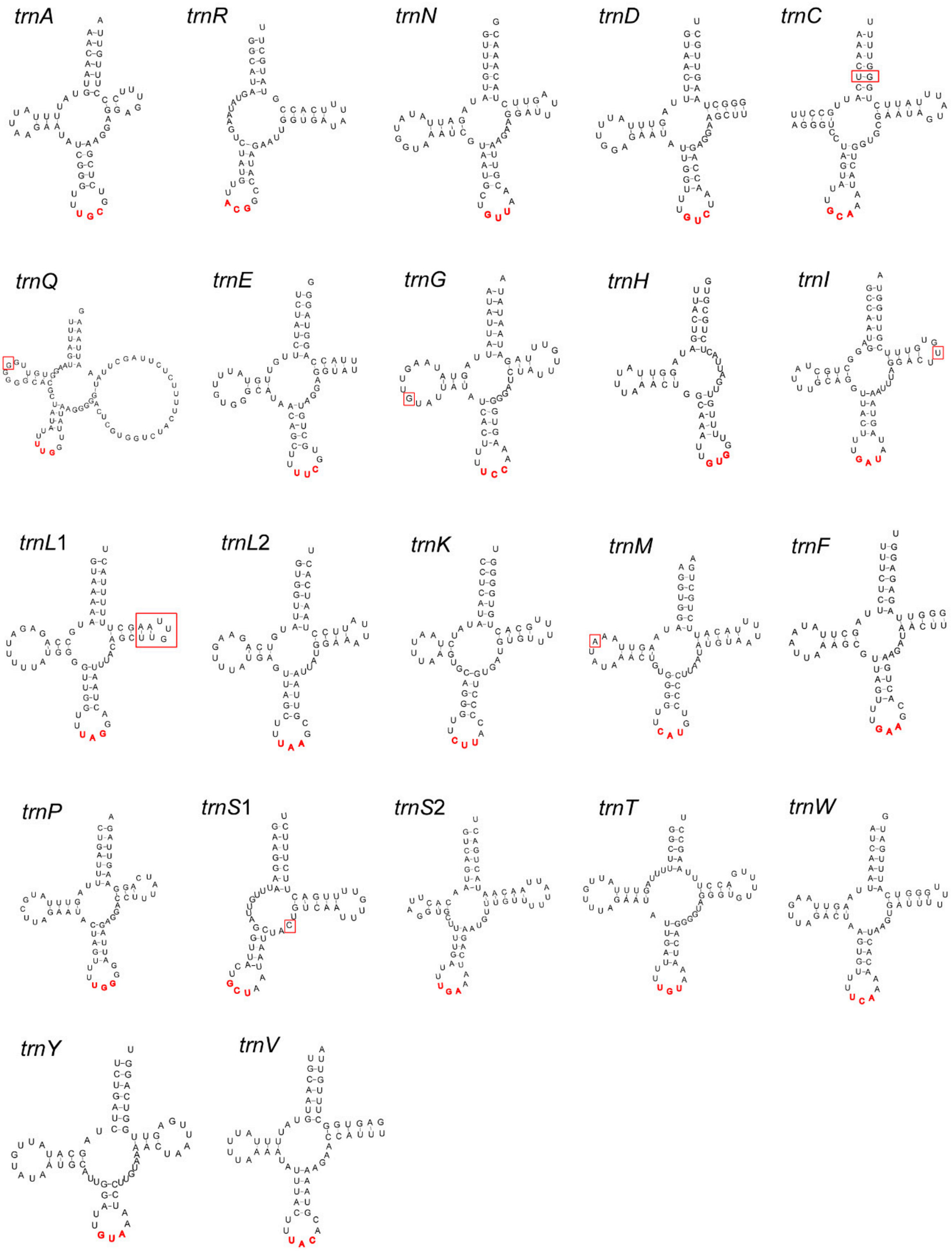

Figure S3 Predicted secondary structures of 22 tRNAs in the *Spirometra mansoni* mitochondrial genome from Anhui, China. Red boxes indicate differences from the Guangxi isolate.

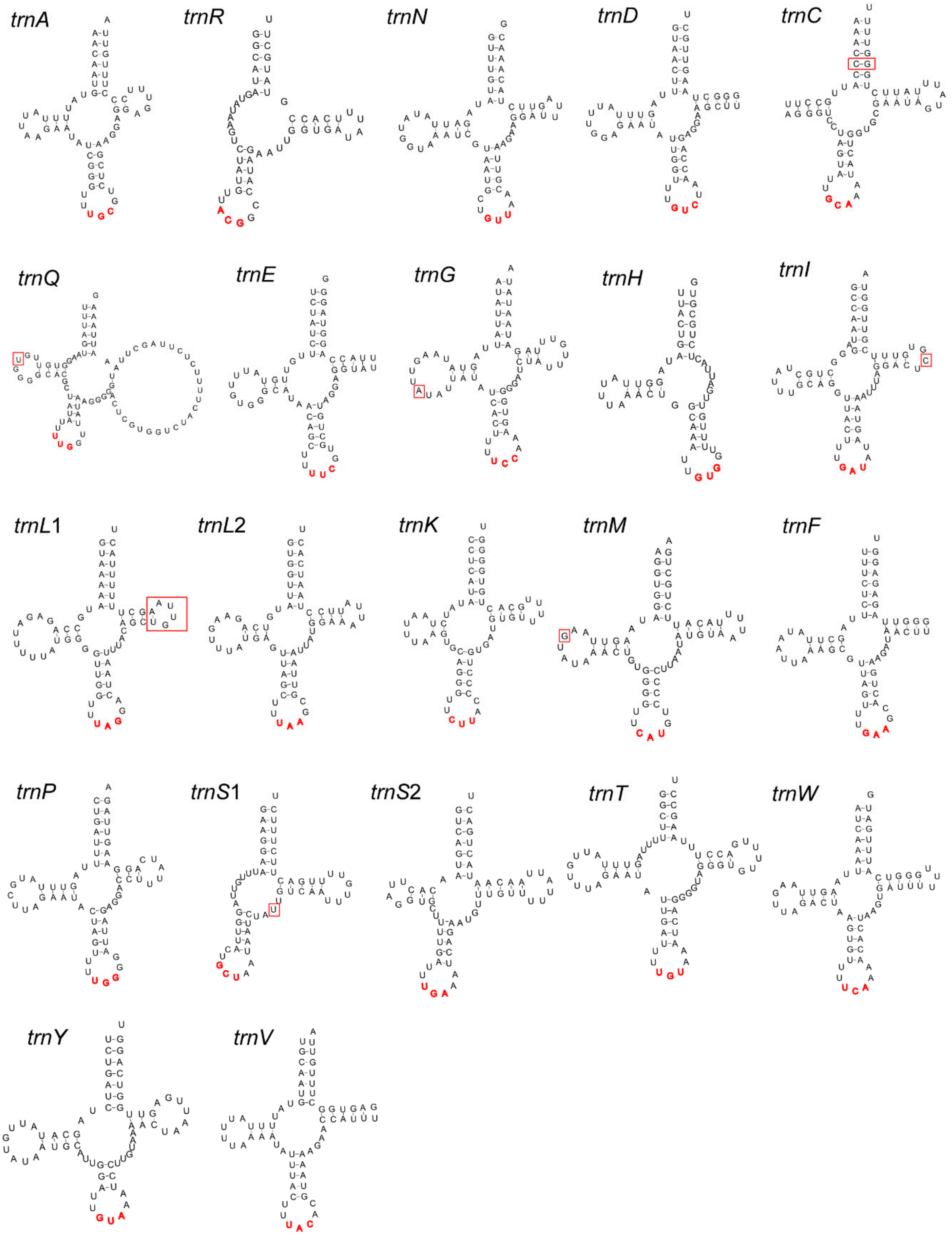

Figure S4 Predicted secondary structures of 22 tRNAs in the *Spirometra mansoni* mitochondrial genome from Guangxi, China. Red boxes indicate differences from the Anhui isolate.

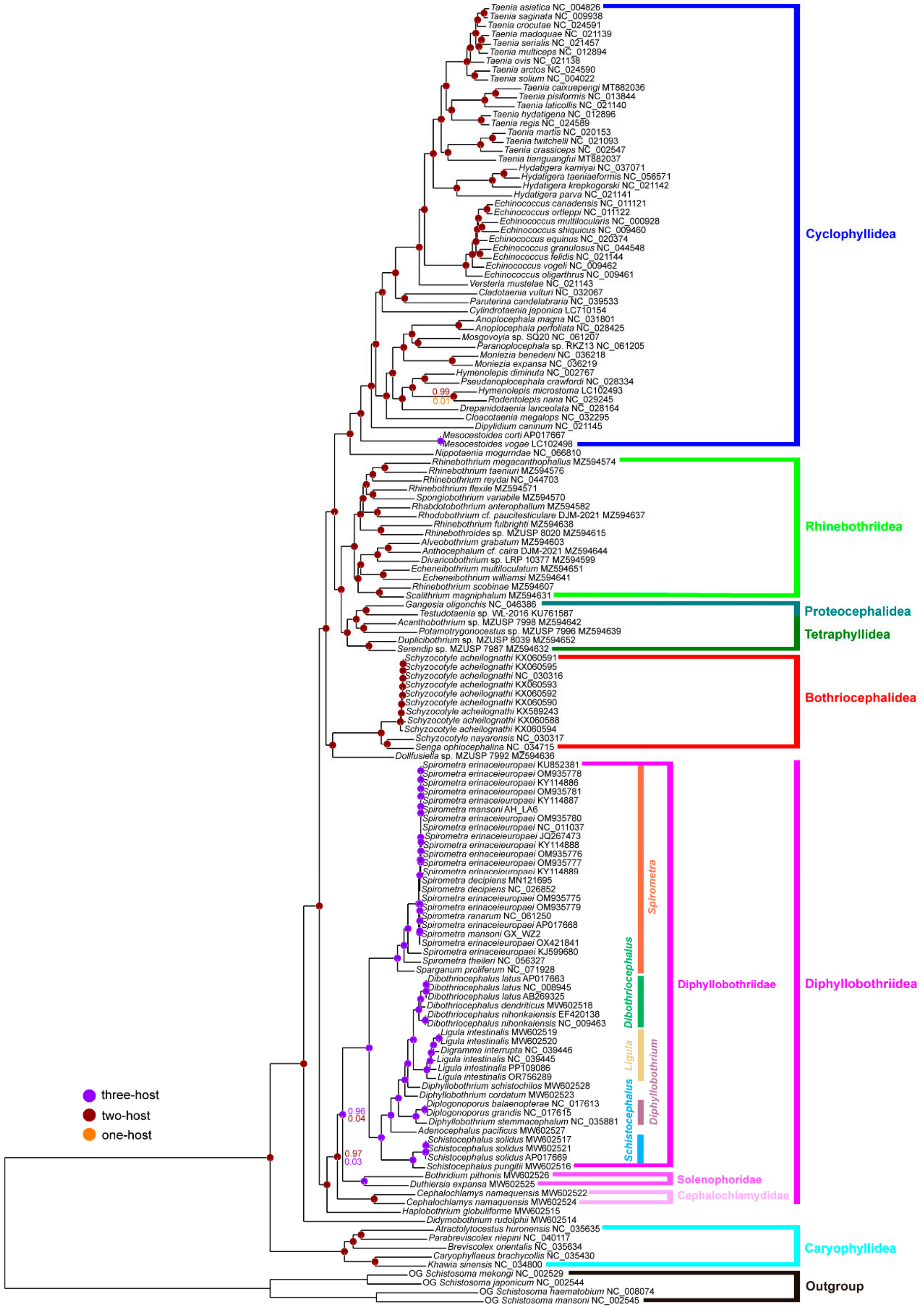

Figure S5 Ancestral reconstruction of life cycle host number in cestodes. Node colours indicate the probability of state occurrence, with numerical values shown only for uncertain nodes (unlabelled nodes have a probability of 1.0).

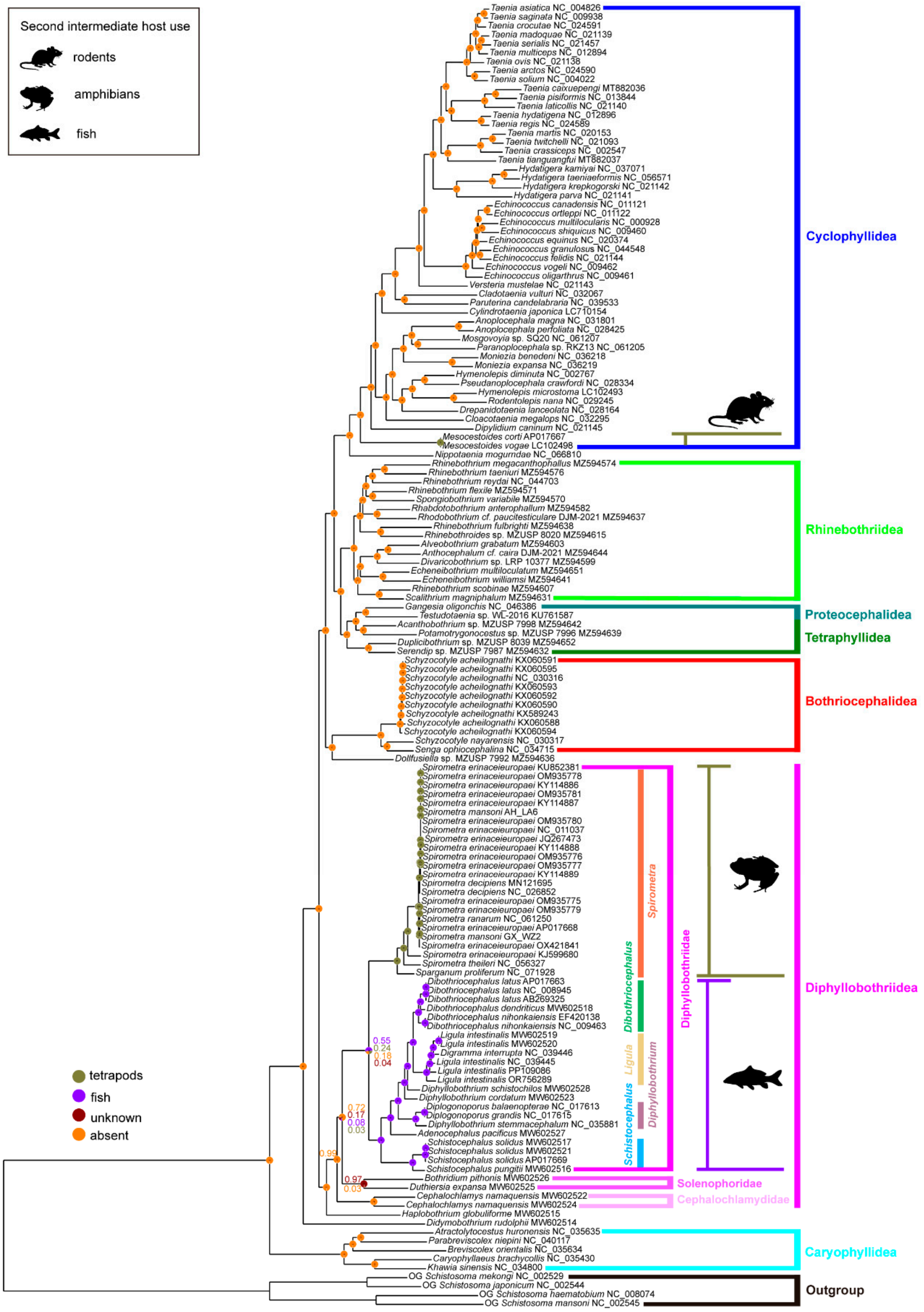

Figure S6 Ancestral reconstruction of second intermediate host types in cestodes. Node colours indicate the probability of state occurrence, with numerical values shown only for uncertain nodes (unlabelled nodes have a probability of 1.0). The second intermediate host type is labelled only for taxa in which it has been clearly identified.
